# The impact of shortening shifts of physicians during their residency on patients and physicians: A systematic review and meta-analysis

**DOI:** 10.1186/s13584-025-00715-2

**Published:** 2025-09-03

**Authors:** Vered Daitch, Itamar Poran, Leonard Leibovici

**Affiliations:** 1 Internal Medicine E, Beilinson Hospital, Rabin Medical Center, 39 Jabotinsky Road, Petah-Tikva, 49100 Israel; 2 Dina Recanati School of Medicine, Reichman University, Herzliya, Israel; 3 Intensive Care Unit, Beilinson Hospital, Rabin Medical Center, Petah-Tikva, Israel; 4 Gray Faculty of Medical & Health Sciences, Tel Aviv University, Ramat Aviv, Israel; 5 Research Authority, Rabin Medical Center, Beilinson Hospital, Petah-Tikva, Israel

**Keywords:** Resident physician shifts, Shift shortening, Duty hours, Work-hour limitations, Shift reforms, Physician safety, Patient safety, Residency quality, Fatigue, Cognitive impairment

## Abstract

**Background:**

Prolonged shifts in residency contribute to physician fatigue, cognitive decline, and increased medical errors. This systematic review and meta-analysis evaluate how reducing shift length affects patient-physician safety, physician well-being, and residency training, addressing the ongoing challenge of balancing resident welfare, patient outcomes, and educational standards across varied implementation settings.

**Methods:**

A comprehensive search of PubMed, EMBASE, The Cochrane Library, Google Scholar, and opengrey.eu was performed from database inception to January 2024. Eligible studies assessed the effects of duty hour limitations (≤ 24 h) on clinical, educational, or systemic outcomes. Both randomized controlled trials and observational studies were included. Meta-analyses used random-effects models. Risk of bias was assessed with RoB 2.0 and ROBINS-I tools. Subgroup analyses were performed by specialty, shift duration, and publication period. Sensitivity analyses excluded studies with extended timeframes.

**Results:**

A total of 108 studies (8 RCTs, 100 observational) were included. Shift shortening was associated with improved resident well-being, including reduced fatigue and work-life balance. Patient safety remained stable, with a significant reduction in 30-day mortality for shifts ≤ 16 h (pooled OR 0.84, 95% CI 0.79–0.89). No significant effect on complications or adverse events was observed. Operative experience showed mixed results, with a non-significant reduction in case volume (pooled std. mean difference 0.65, 95% CI -0.04 to 1.34, *P* = 0.07), while test scores exhibited minimal changes. Effect directions remained consistent across publication periods. High heterogeneity and risk of bias were observed across most included studies.

**Conclusions:**

Shortening shifts to 24 h or less appears to improve residents’ satisfaction and work-life balance while maintaining patient safety outcomes. Educational outcomes were mixed; operative experience was preserved in some settings, while effects on non-surgical training remain less clear. These findings underscore the importance of tailoring reforms to specialty needs and training contexts. Future research should examine unstudied outcomes, such as residency attrition or shifts to less demanding specializations, and system-wide implementation costs. A stepped wedge cluster randomized trial is recommended for future policy evaluations.

**Systematic review registration:**

PROSPERO CRD42023390197

**Supplementary Information:**

The online version contains supplementary material available at 10.1186/s13584-025-00715-2.

## Introduction

Fatigue impairs attention, decision-making, and task performance across settings. In healthcare, prolonged shifts (> 12 h) have been associated with impaired cognitive performance and increased risk of medical errors [1–4]. In response, an ongoing effort over the past two decades has sought to reduce residency shift lengths to mitigate these risks. In 2003, the US Accreditation Council for Graduate Medical Education (ACGME) set a 24-hour limit on resident work hours, with an option for a 6-hour extension for specific duties (ACGME 2003-DHR) [5]. In 2008, the US National Academy of Medicine recommended a 16-hour limit, partially adopted in 2011 (ACGME 2011-DHR) [6, 7] but later rescinded following evidence indicating disruptions in team continuity, increased handoffs, and restricted scheduling flexibility [8, 9]. In Europe, the European Working Time Directive (EWTD), which set a 48-hour weekly limit with defined rest periods, was enforced in the healthcare sector starting in 2009 [10]. While these changes were intended to improve working conditions and patient-physician safety, concerns have arisen about their impact on residency quality and continuity of care.

Systematic reviews on shift-shortening reforms reveal mixed outcomes: Awan et al. reported no significant improvement in patient outcomes, a reduction in surgical experience for junior residents, and a shift in clinical responsibilities to senior residents [11]. Rodriguez-Jareño et al. linked extended working hours to increased percutaneous injuries and road traffic accidents among residents, though findings on mood disorders and general health were inconclusive [12]. The impact of these reforms has varied widely across different medical settings and specialties, highlighting the ongoing debates regarding their effectiveness [13–17]. 

While duty hour reforms were intended to improve physician and patient safety, their overall impact—particularly on training quality and care continuity—remains incompletely understood. Earlier reviews often focused on isolated outcomes or specific specialties [11–17], and many predate recent studies evaluating updated scheduling models such as night float and split shifts. Our review provides an integrated synthesis across shift durations (13, 16, and 24 h), specialties, and outcome domains—including patient and resident safety, resident well-being, and education—offering timely insights as health systems re-evaluate post-pandemic workforce policies. This international perspective was also motivated by local reforms in Israel, where resident shifts were recently reduced from 26 to 19 h in selected hospitals, underscoring the urgency of updated, evidence-based guidance for such policy transitions.

Accordingly, the aim of this review is to systematically evaluate the effects of shift-shortening interventions on patient safety, resident safety and well-being, and educational outcomes during residency training.

## Methods

Following PRISMA guidelines [18], a systematic review and meta-analysis were conducted on studies from inception to January 2024. Data sources included PubMed, EMBASE, The Cochrane Library, Google Scholar, and open grey.eu. For the full search phrase see Supplemental Appendix 1.

### Study selection

Eligible studies included cluster randomized trials (CRT), randomized controlled trials (RCTs), non-randomized trials, observational comparison studies (including concomitant cohort studies, historical controls, and case-control studies), as well as before-after studies conducted within the same participants or institutions.

### Intervention and comparison

Shift shortening is defined as reducing shift lengths below previously established norms, primarily encompassing 24-hour shifts (ACGME 2003-DHR), 16-hour shifts (ACGME 2011-DHR), and 12-13-hour shifts (EWTD). The comparison group maintained standard shifts (≥ 24 h).

### Outcomes

Primary outcomes included patient safety (30-day mortality, complications, adverse events), physician safety and well-being (stress, anxiety, depression, burnout, accidents, injuries, substance abuse), and residency quality (operative volume, test scores). Secondary outcomes encompassed patient care (continuity, quality, satisfaction), physician outcomes (attrition rates, quality of life, satisfaction), and changes in cost and staffing.

### Data extraction and analysis

Data were extracted independently by two investigators. Risk of bias was assessed using RoB 2 (for RCTs) and ROBINS-I (for non-randomized studies) [19, 20]. Meta-analysis was conducted using RevMan 5.4, employing random-effects models for significant heterogeneity (I^2^ > 50%). Odds ratios (OR) and risk ratios (RR) were calculated where applicable. Pooled ORs with 95% confidence intervals (CIs) were computed for patient safety outcomes, including 30-day mortality, complications, and adverse events. Pooled Risk Ratios (RRs) were calculated for RCT-specific analyses. Studies lacking event data or sample size details were not included in the pooled analysis and were presented descriptively. Sensitivity analyses excluded trials exceeding five years to minimize confounding effects from evolving medical practices and patient characteristics [21–24]. Subgroup analyses were performed based on specialty and shift duration to address clinical heterogeneity. Standardized mean differences with 95%-CIs were calculated for residents’ educational outcomes, specifically operative experience. Studies missing key statistical measures were analyzed descriptively. Separate pooled analyses were performed for patient safety and resident experience to assess the impact of shift shortening to ≤ 16 h. To explore whether effect patterns changed over time, we performed subgroup analyses stratified by 5-year publication periods. Forest plots of these analyses are provided in Supplementary Figs. 1–4. Since only crude published data were used, adjusted analyses could not be conducted.

## Results

**Fig. 1:**
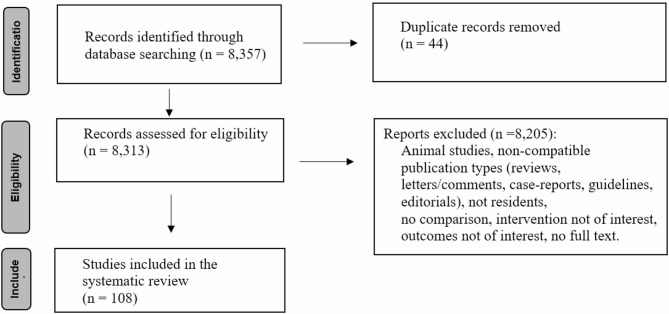
PRISMA flow diagram. This figure illustrates the flow of information through the different phases of the systematic review. It maps out the number of records identified, included and excluded, and the reasons for exclusions

From 8,357 screened publications, 108 studies (including 8 RCTs) were included (Fig. 1) [1, 25–135].

The primary interventions analyzed were 24-hour shifts (ACGME 2003-DHR, 55/108), 16-hour shifts (ACGME 2011-DHR, 29/108), and 13-hour shifts (EWTD, 10/108). Of these, 47 studies primarily examined patient safety, while 35 assessed residency training quality. No studies investigated residency attrition rates or shifts to less demanding specializations. Table 1 summarizes the characteristics of the included studies by country, study design, type of specialty, and intervention type. For a more detailed description of each study’s aims, methods, setting, and findings, see Supplementary Table 1.

**Table 1:** Characteristics of the included studies (*N* = 108)

Characteristic	*N* (%)
Country	
United States	84 (78)
Canada	5 (5)
Europe (Germany, The Netherlands, Ireland, Switzerland, France)	6 (6)
United Kingdom	6 (6)
Asia (Japan, Israel, Korea, Pakistan, Hong Kong, Saudi Arabia, Jordan)	7 (6)
Type of center	
Nationwide	27 (25)
Single center	62 (58)
Multicenter	19 (17)
Study design	
Randomized controlled trial	8 (7)
Cohort study (concomitant or with historical controls)	42 (39)
Retrospective or cross-sectional study	43 (40)
Survey	15 (14)
Type of residency*	
Mixed	14 (13)
Surgical	48 (44)
Mixed	15 (14)
General surgery	19 (18)
Orthopedic	5 (5)
Neurosurgery	4 (4)
Cardiothoracic surgery	4 (4)
ENT	3 (3)
Plastic surgery	1 (1)
Pediatric	14 (13)
Obstetrics and Gynecology	9 (8)
Medical	23 (21)
Other (Family medicine, anesthesia)	3 (3)
Type of shortening	
European working time directive 1993 (up to 13 consecutive hours)	10 (9)
Accreditation Council for Graduate Medical Education 2003 (up to 24 consecutive hours)	55 (51)
Accreditation Council for Graduate Medical Education 2011 (up to 16 consecutive hours)	29 (27)
Both 2003 and 2011 Accreditation Council for Graduate Medical Education	3 (3)
Other (12 h, 14 h, 18 h, 12 h vs. 16 h vs. 24 h)	12 (11)
Outcomes*	
Type of outcome for patients	
Safety	48 (44)
Quality of care	4 (4)
Patients’ satisfaction	2 (2)
Type of outcome for residents	
Safety	14 (13)
Quality of residency	35 (32)
Residents’ satisfaction and QOL	20 (19)

N (%) indicates the number of studies and the percentage they represent out of the total 108 studies reviewed

Cumulative percentages may exceed 100% due to some categories overlapping across multiple study characteristics

‘Mixed’ under ‘Type of residency’ includes studies that examined surgical and medical specialties

‘Type of shortening’ refers to the maximum shift lengths permitted under different regulatory frameworks or study conditions

### Patient outcomes

#### 30-day mortality

**Fig. 2:**
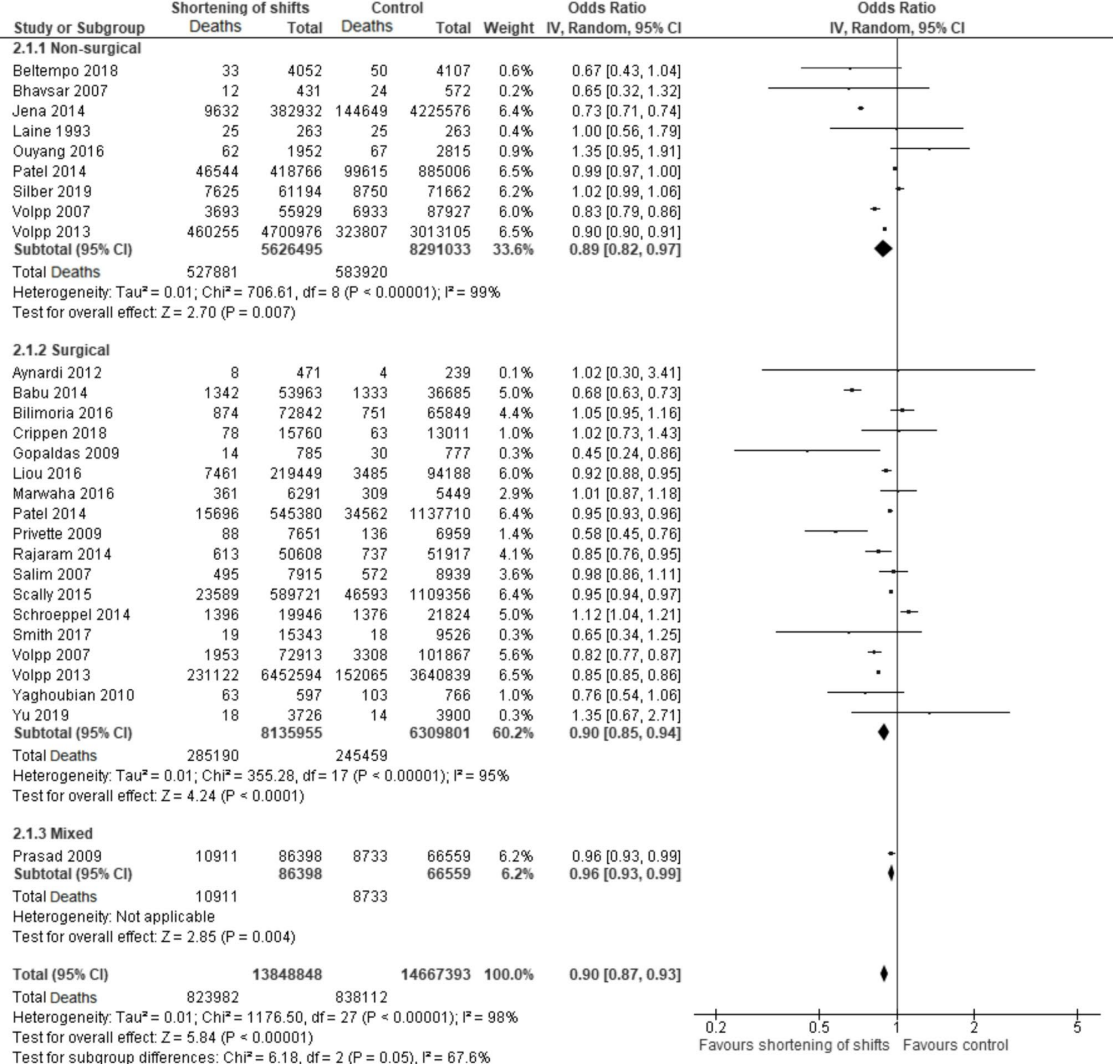
Forest plot describing the association of shift shortening and patient 30-day mortality. This forest plot displays the association between shortened shifts for medical residents and patient 30-day mortality rates across various studies

Twenty-five studies were included in this comparison [25–49], featuring two RCTs [48, 49]. In non-surgical and in surgical specialties there was an association between interventions for shortening of shifts and reduced 30-day mortality (pooled OR 0.89, 95% CI 0.82–0.97 and pooled OR 0.89, 95% CI 0.85–0.94, respectively), with significant heterogeneity (I^2^ = 98%) (Fig. 2).

**Fig. 3:**
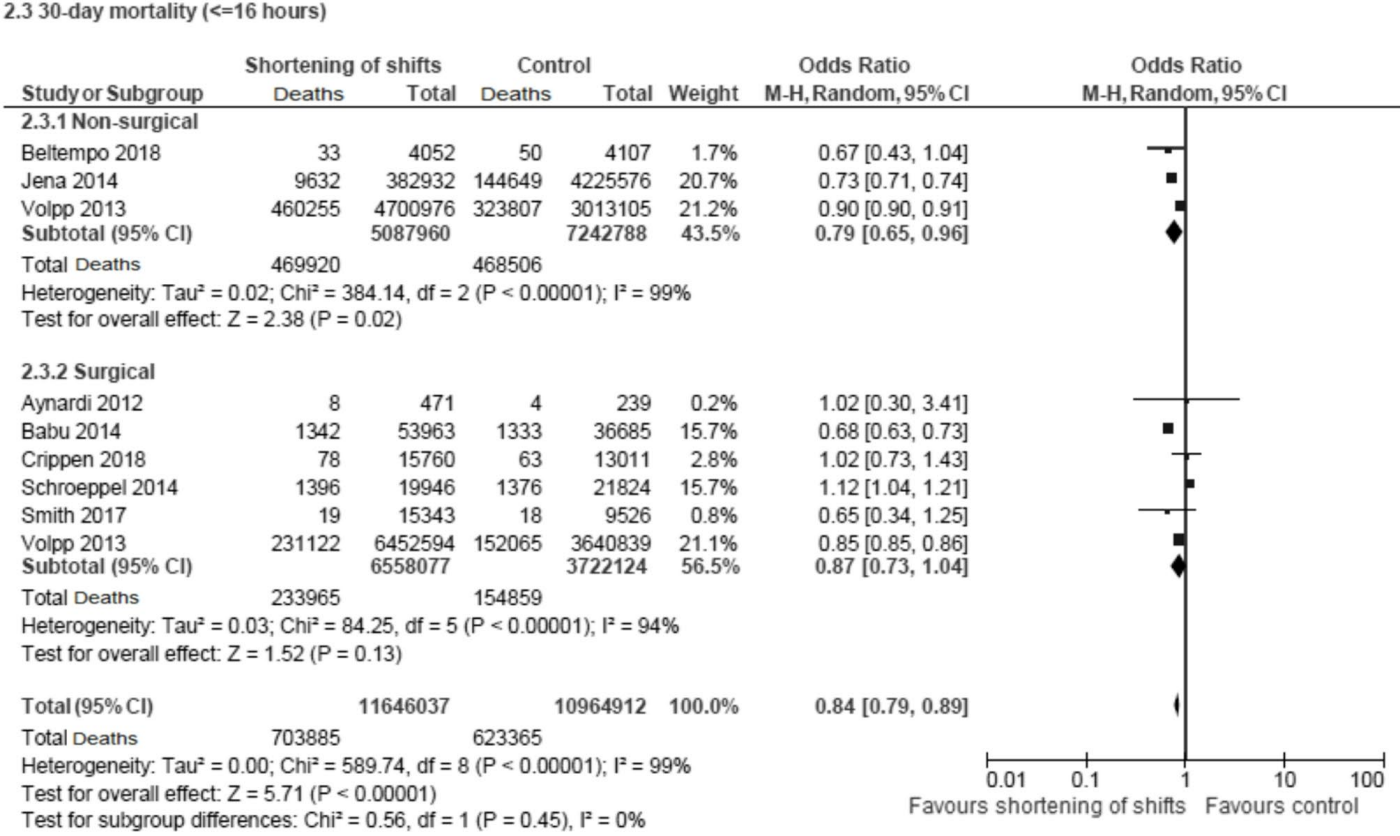
Forest plot describing the association of shortening of shifts to 16 h or less and patient 30-day mortality. This forest plot specifically focuses on the impact of limiting shifts to 16 hours or less on patient 30-day mortality

A sensitivity analysis excluding eleven studies, in which the study period was longer than 5 years, reduced heterogeneity to 73% and altered the association in non-surgical specialties to non-significant (pooled OR 1.00, 95% CI 0.96–1.05). RCTs showed no significant impact (pooled RR 1.02, 95% CI 1.00-1.05). However, shifts ≤ 16 h were associated with significantly reduced mortality (pooled OR 0.84, 95% CI 0.79–0.89) (Fig. 3).

An exploratory subgroup analysis by publication period (before 2010, 2010–2014, 2015–2019, and 2020 and beyond) is presented in Supplementary Figs. 1–2. The direction of effect remained consistent across all time periods.

Six studies on patient mortality including non-surgical and surgical specialties were excluded from the pooled analysis [50–55]. Among them, three found no association between shift shortening and mortality [51, 52, 54] two indicated reduced mortality rates in teaching hospitals after ACGME 2003-DHR implementation [50, 55], and one suggested a negative association between DHR and mortality in patients with nervous system pathology [53]. 

#### Complications and adverse events

**Fig. 4:**
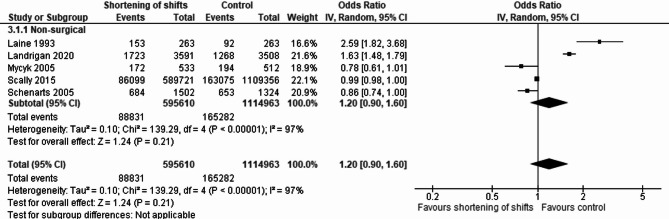
Forest plot describing the association of shift shortening and non-surgical complications and adverse events. This forest plot displays the association between shift shortening and the occurrence of non-surgical complications and adverse events in patients

**Fig. 5:**
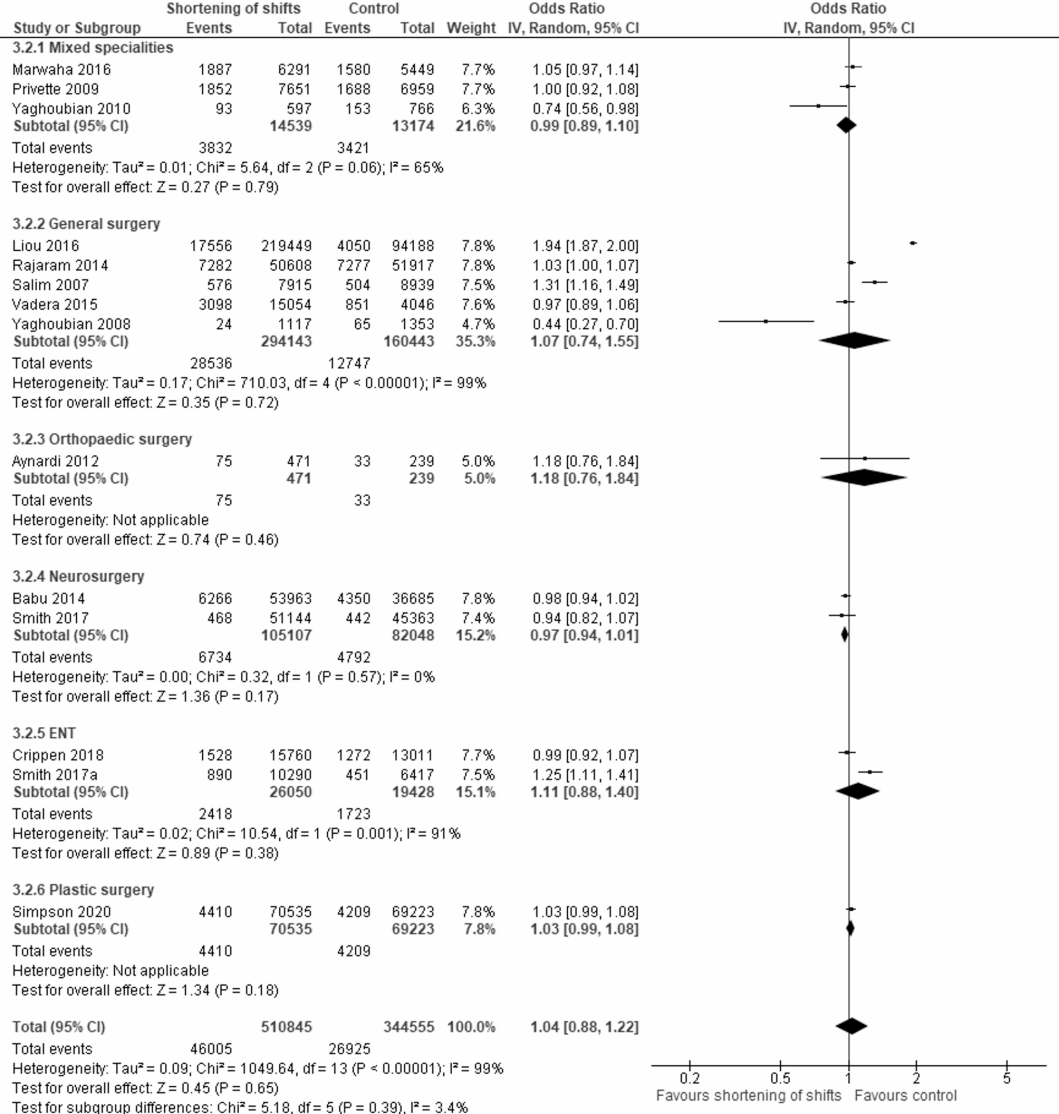
Forest plot describing the association of shift shortening and surgical complications and adverse events per type of specialty. This forest plot displays the association between shift shortening and the occurrence of surgical complications and adverse events in patients, across various medical specialties

Performing separate analyses for non-surgical and surgical specialties, we included five studies in the non-surgical comparison [32, 46, 54–56], of which one is an RCT and fourteen studies in the surgical comparison [26, 28, 30, 31, 33, 37, 39–41, 49, 57–60]. No significant association was found between shift shortening and complications or adverse events in both non-surgical (pooled OR 1.20, 95% CI 0.90–1.60) (Fig. 4) and surgical specialties (pooled OR 1.04, 95% CI 0.88–1.22) (Fig. 5), with notable heterogeneity.

Eleven studies examining patient complications and adverse events including non-surgical and surgical specialties were excluded from the pooled analysis. Among these, five studies [48, 61–64] (including two RCTs) [48, 61] found no association between shift shortening and complications, while two studies reported worsened complication rates after the implementation of ACGME 2003-DHR [66, 67]. Additionally, one prospective cohort study and two RCTs suggested an association between shift shortening and decreased Serious Medical Errors (SME) and physician-related SMEs in intensive care units [1, 68, 69].

A separate analysis of studies examining shortening shifts to 16 h or less also showed no significant association, in both surgical and non-surgical specialties (pooled OR 1.02, 95% CI 0.98–1.06, and pooled OR 1.35, 95% CI 0.89–2.04, respectively).

The findings were consistent across different publication periods (Supplementary Fig. 3).

#### Quality of care and satisfaction

In a randomized controlled trial comparing shortening compliant with ACGME 2003-DHR and ACGME 2011-DHR, the latter was associated with a decline in perceived quality and continuity of patient care, evident in increased handoffs, reduced availability for teaching conferences, and decreased daytime presence [70]. Five observational studies explored patient quality of care and satisfaction. Arora et al. found a decrease in patient-reported resident involvement from 20% before 2003 to 12% after 2011, and Maxwell et al. reported that fewer residents followed up on operative cases after the implementation of EWTD [71, 72]. Rajaram et al. concluded that resident duty hour reform did not significantly affect patient experience or process-of-care measures, and Salgado et al. found no significant differences in patient perceptions but noted challenges for interns on extended schedules, such as unfamiliarity with their assigned patients, decreased ability to conduct physical exams on new patients, and decreased quality of teaching [33, 51]. 

### Residents outcomes

#### Operative experience

**Fig. 6:**
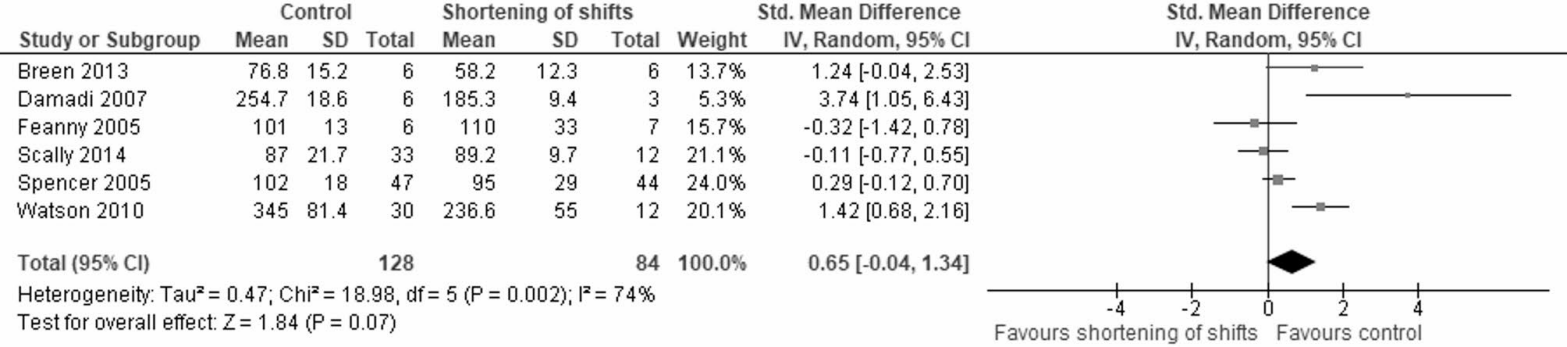
Forest plot describing the association of shortening of shifts and operative experience in general surgery residents (measured as mean number of operations per resident). This forest plot displays the association between shift shortening and operative experience of general surgery residents, measured as the mean number of operations per resident

Six studies focusing on changes in the operative experience of general surgery residents were analyzed [73–78]. There was a non-significant association between interventions for shortening of shifts and reduction of average operative volume (pooled std. mean difference 0.65, 95% CI -0.04 to 1.34, *P* = 0.07), with significant heterogeneity (I^2^ = 74%) (Fig. 6).

Stratifying the analysis by publication period yielded similar findings, with no significant shift in effect direction over time (Supplementary Fig. 4).

Twenty studies examining operative experience in other surgical specialties were excluded from the pooled analysis. Among them, ten studies reported a decrease in operative experience [78–87], eight found no statistically significant differences before and after the implementation of shift shortening [88–95], and three indicated an increase in operative case volume [96–98]. 

#### Test scores

Six studies assessed the effect of shift shortening on residents’ test scores, including the American Board of Surgery In-Training Examination (ABSITE); Council on Resident Education in Obstetrics and Gynecology (CREOG); In-training examination in Internal Medicine organized by the American College of Physicians; basic science; clinical management. In most cases, no statistically significant differences were observed before and after implementing shift-shortening measures [33, 97, 99, 100], including one RCT [100]. However, two studies reported varied outcomes: one noted improved test scores [89], while another identified a minor decrease [86]. 

#### Other educational outcomes

Desai et al. observed no significant alteration in direct patient care time for internal medicine residents following the implementation of shift-shortening measures [100]. Another RCT by Desai et al. within the field of internal medicine reported diminished availability for teaching conferences and reduced daytime presence [70]. Auger et al. studied the impact of ACGME 2011-DHR compliance on resident education, reporting a significant decrease in resident presentations on rounds without affecting interactions with faculty or perceived learning [101]. Johnson et al. reported a significant increase in research publications by orthopedic residents after the implementation of ACGME 2011-DHR [102]. No significant differences were found in the acquisition of theoretical knowledge among PICU residents after shift shortening [103]. 

#### Safety outcomes

Fourteen studies, including six RCTs, examined safety outcomes among residents [60–62, 69, 70, 106–114]. Among the RCTs, Rahman et al. found lower rates of attentional failure and sleepiness in pediatric residents with shorter shifts [60]. Parshuram et al. found no significant differences in overnight sleepiness, fatigue, and burnout among internal medicine residents with different schedules [61]. Desai et al. reported increased sleep duration during on-call periods for internal medicine residents on shorter shifts [70]. Huber et al. found no discernible difference in fatigue levels between pediatric residents on shorter or longer shifts [106]. Persico et al. observed cognitive alterations after a 24-hour shift but not after a 14-hour night shift for emergency physicians [107]. Basner et al. reported no disparities in chronic sleep loss, sleepiness, or alertness between residents on shortened or extended shifts [108]. 

Non-randomized trials showed mixed results: one study highlighted higher attentional failures during traditional night shifts compared to shortened ones [110]. Two studies reported comparable sleepiness levels regardless of shift length [109, 112]. Schumacher et al. observed reduced sleepiness during driving from work [113]. Nomura et al. discovered consistent depressive symptoms before and after shift changes [111]. Following shift-shortening, more residents with over 8 patients experienced burnout, although overall rates did not show significant differences [109]. 

Weaver et al. evaluated the impact of ACGME 2011-Duty Hour Regulations on first-year resident physicians. They found that reduced duration shifts and shortened weekly work hours were associated with decreased risks: motor vehicle crashes decreased by 24%, percutaneous injuries by over 40%, and attentional failures by 18% [114].

#### Satisfaction and quality of life (QOL)

Four RCTs explored residents’ satisfaction and quality of life [48, 103, 106, 107]. Barger et al.‘s survey of 290 resident physicians revealed no significant differences in skill and knowledge acquisition or negative impact on daily activities between Extended-Duration Work Rest (EDWR) (≥ 24 h. shifts) and Regular Clinical Work Rest (RCWR) rotations (≥ 16 h. shifts). RCWR rotations, however, led to more negative ratings of work experience and educational quality, with residents obtaining significantly more weekly sleep during RCWR [103]. Bilimoria et al. found flexible-policy residents (PG1 > 16 h. shifts) were more dissatisfied with rest time and perceived negative effects on factors related to time away from the hospital compared to standard-policy residents (PG1 ≤ 16 h. shifts), but there were no significant differences in job satisfaction, career choice satisfaction, morale, or fatigue affecting safety [48]. Huber et al. observed no significant differences in perceived knowledge, professionalism, or fatigue levels between call (up to 28 h) and shift (12-hour shifts) schedules [106]. Persico et al. found no statistically significant correlations between cognitive performance outcomes and self-assessed fatigue levels, attention, mood degradation, and sleep quality among emergency physicians following 14-hour and 24-hour shifts [107]. 

Sixteen observational studies investigated the impact of shortening resident work hours on well-being, healthcare quality delivery, and medical education experience. These studies indicated that reducing work hours improved residents’ personal life satisfaction while maintaining stable job satisfaction. Shorter shifts positively influenced sleep quality, reduced smoking rates, and increased life satisfaction, correlating with better sleep and lower anxiety and depression scores. Importantly, these changes did not significantly undermine residents’ overall educational experience [25, 56, 115–128]. 

### Risk of bias assessment

This systematic review and meta-analysis analyzed eight RCTs, mostly cluster-randomized, alongside 100 observational studies. Seven RCTs were identified as having a ‘high risk of bias’, while one trial raised ‘some concerns of bias’, mainly due to limitations such as the absence of intention-to-treat analysis, inadequate information on missing data, and lack of blinding, including the outcome assessor (Supplemental Table 2).

The observational studies, constituting a substantial portion of the studies examined, demonstrated predominantly a high risk of bias (95%) (Supplemental Table 3). This high risk can be attributed, in part, to the prevalence of extensive cohort studies and before-after analyses featuring distinct participant groups over varying time frames.

## Discussion

This comprehensive systematic review and meta-analysis, comprising 108 studies including eight RCTs, investigated the impact of shift-shortening interventions in medical practice. Shifts primarily shortened to 24, 16, and 13 consecutive working hours were evaluated. The diversity in training models and the implementation of these interventions across different medical specialties provide a broad perspective on their effects. Patient safety outcomes revealed a significant reduction in 30-day mortality across surgical and non-surgical specialties with shortened shifts. However, this effect diminished when longer-term non-surgical studies were excluded. This reduction in effect highlights the impact of confounding factors and potential biases in longer studies. For instance, changes in medical practice or technology, variations in patient demographics, and shifts in healthcare policies may influence these results [20–23]. Additionally, reporting practices can evolve, potentially altering the documented rates of adverse events [24]. 

RCTs did not show significant impact on 30-day mortality. Overall, shift-shortening interventions did not significantly impact complications or adverse events, suggesting their safety for patients.

Patient quality of care and satisfaction showed mixed results, with residents perceiving a decline in care continuity but no significant differences observed in patient satisfaction. Resident outcomes indicated improved satisfaction with personal life and work-life balance with shorter shifts, alongside better sleep quality, reduced smoking rates, and increased life satisfaction. Safety outcomes among residents showed decreased accidents and attentional failures with shorter shifts. Study findings underscored the complex effects of shift-shortening measures on medical education and residency quality. While some studies indicated a reduction in operative experience, others showed no significant differences or even an increase in case volume, suggesting careful planning can preserve operative experience despite shift duration alterations. However, the inconsistency highlights the need for further research to determine what factors can influence the operative experience across surgical specialties. In non-surgical education, reductions in resident presentations were noted, but increased research publications were observed, with consistent academic performance despite altered work schedules.

The impact of shortening shifts on training varies significantly across different medical specialties, reflecting diverse training requirements and clinical demands. For example, surgical training programs, which require extensive hands-on experience and continuity of care, may face challenges with shortened shifts, whereas non-surgical specialties might adapt more easily. This variation underscores the necessity of specialty-specific evaluations when implementing shift changes. To better account for this variation, subgroup analyses by specialty type and shift duration were conducted, allowing for more accurate interpretation within comparable clinical contexts despite the overall heterogeneity. It also highlights the importance of contextual factors, including the nature of the specialty and the design of the residency program, in assessing the differential impact of shift length adjustments. These variations also pose broader challenges for health systems implementing shift reforms. Successful adoption likely depends on aligning scheduling changes with program characteristics, staffing capacity, and local resource constraints, including costs, supervision structures, and potential extension of training duration.

Our findings, aligning with prior research [13–17], indicate that shorter shift interventions have not significantly impacted patient safety outcomes. Earlier reviews have also noted enhancements in residents’ quality of life, sleep patterns, and reduced fatigue due to such interventions [13, 14, 16, 17]. However, findings related to medical education and residency quality, similar to our study, have varied in other reviews. A 2010 systematic review examining the reduction of resident shifts to 16 h indicated generally unchanged medical education [14]. In contrast, a 2014 systematic review reported a perceived negative impact on surgical operative and technical skills, with conflicting evidence on resident education [16]. Two subsequent reviews from 2015 to 2016, found adverse effects on resident education [13, 15]. 

The study has limitations that influence the interpretation of results. There is considerable heterogeneity of interventions and outcomes, reliance on subjective evaluations for resident well-being, and a predominant focus on surgical trainees in educational studies. The included studies did not assess long-term educational outcomes or residency attrition rates, and the complexity of interventions makes it difficult to solely attribute changes in outcomes to the reduction in shift duration.

Most included studies focused on high-volume specialties such as internal medicine and general surgery, with limited data from smaller fields like ENT or neurology. This underrepresentation may affect the generalizability of our findings across all specialties.

In terms of geographic distribution, the majority of studies were conducted in the United States, reflecting its early and sustained focus on duty hour regulation. Although residency models differ internationally, many high-income countries maintain comparable hospital-based training systems, allowing for cautious extrapolation of these findings while acknowledging differences in health system context.

A predominant number of reviewed studies and trials display a high risk of bias. It is acknowledged that, especially in the context of observational studies examining policy outcomes, the preferred study design often leans towards before-after designs. Some of the RCTs in the review exhibit cross-over designs, which may elevate the risk of bias. Directly comparing 16–24-hour shifts to longer durations in contemporary settings raises legitimate ethical concerns, especially given evolving standards for resident safety. In light of these concerns, a stepped wedge cluster randomized trial may offer a more ethical and pragmatic approach. In this design, different clusters (e.g., hospitals or departments) transition from control to intervention at randomized time points. This enables gradual, staged implementation across settings, while allowing valid comparisons both within and between clusters [136]. 

## Conclusions

Shortening shifts to 24 h or less emerged as a positive factor, improving residents’ well-being and safety without compromising patient outcomes. However, the effects on medical education were equivocal. These findings underscore the importance of tailoring reforms to the specific needs of each specialty and training environment, supported by system-level planning and adequate resources. Given the high heterogeneity and overall risk of bias in many included studies, findings should be interpreted with caution. Future research should focus on underexplored outcomes—such as the effects on non-surgical training, residency attrition, and career trajectory—as well as on the organizational and financial implications of shift reforms.

### Policy implications and recommendations

In Israel, a 2023 pilot introduced 19–21-hour shifts in selected non-surgical departments in peripheral hospitals. International experience highlights common barriers, including staffing shortages and resistance from professional groups. For instance, duty hour reforms in the U.S. and U.K. raised concerns regarding supervision and the quality of training [8, 11]. Better outcomes were observed when local clinical workflows were considered, stakeholders were engaged early, and phased rollouts were planned [13]. Successful implementation depended on parallel strategies to maintain training quality and continuity of care, such as structured learning during non-clinical hours, enhanced supervision, and standardized handovers [3, 56]. However, early experience from Israel and other countries suggests that insufficient staffing, limited funding, and inadequate supervisory capacity can undermine reform efforts, sometimes leading to reversion to longer shifts [70, 99]. In the Israeli pilot, reports indicated that some hospitals reverted to 24-hour shifts due to these challenges. Such outcomes highlight three critical barriers: (a) workforce shortages and the limited number of residents available nationally, (b) the need for sustainable financial investment, and (c) the challenge of adapting reforms to the diverse clinical and educational demands of different specialties.

To address these barriers, particularly in the context of the Israeli reform, policymakers should secure long-term funding for additional residency positions, offer targeted incentives to attract and retain residents in underserved areas, redistribute workload across Internal Medicine and other specialties, and design specialty-specific implementation strategies to safeguard training quality and patient care [14–16]. These measures should be implemented gradually, alongside robust evaluation frameworks, such as a stepped-wedge cluster randomized trial, to monitor outcomes meaningful to patients, residents, and the health system.

## Supplementary Information

Below is the link to the electronic supplementary material.

Supplementary Material 1

Supplementary Material 2

Supplementary Material 3

Supplementary Material 4

Supplementary Material 5

Supplementary Material 6

## Data Availability

All data analyzed in this study are publicly available within the published literature. The dataset generated and analyzed during the current study is included in the article and its supplementary materials.

## Abbreviations

CI: Confidence Interval
PRISMA: Preferred Reporting Items for Systematic Reviews and Meta-Analyses
