# Supplementary material for: The impact of shortening shifts of physicians during their residency on patients and physicians: A systematic review and meta-analysis
Source: Isr J Health Policy Res. 2025 Sep 3;14:53. doi: 10.1186/s13584-025-00715-2 (PMC12406601; doi:10.1186/s13584-025-00715-2)
Supplement: Supplementary file 1 — Supplementary Material 1 [file 13584_2025_715_MOESM1_ESM.docx]

**Supplemental Appendix 1- Search strategy:**

Shortening of shifts:

1. Short*
2. Restrict*
3. Reduc*
4. Limit*
5. OR/1-4
6. “Duty hours”
7. “Working time”
8. “Working hours”
9. “Work schedule”
10. shift*
11. Workload [MeSH]
12. OR/4-11
13. #4 AND #12

((Short* OR Restrict* OR Reduc* OR Limit*) AND ("Duty hours" OR "Working time" OR "Working hours" OR "Work schedule" OR shift* OR Workload [MeSH])): 159,754 results.

Interns and resident physicians:

1. Personnel Staffing and Scheduling [MeSH]
2. Medical Staff, Hospital [MeSH]
3. Internship and residency [MeSH]
4. Training
5. Fellow*
6. Intern*
7. Medical resident*
8. Resident physician*
9. OR / 14 – 21
10. Nurse*
11. Hospital end
12. #22 NOT #23

(((Personnel Staffing and Scheduling [MeSH]) OR (Medical Staff, Hospital [MeSH]) OR (Internship and residency [MeSH]) OR Training OR Fellow* OR Intern* OR (Medical resident*) OR (Resident physician*)) NOT Nurse*): 4,894,480 results.

Study design:

1. Cohort stud*
2. Cohort studies [MeSH]
3. Cross-sectional studies [MeSH]
4. Epidemiologic stud*
5. Epidemiologic studies [MeSH]
6. Longitudinal stud*
7. Observational stud*
8. Population stud*
9. Prospective stud*
10. Prospective studies [MeSH]
11. Retrospective stud*
12. Retrospective studies [MeSH]
13. Randomized controlled trial [Publication Type]
14. Controlled clinical trial [Publication Type]
15. Randomized [Title/Abstract]
16. Randomly [Title/Abstract]
17. Trial [Title/Abstract]
18. Groups [Title/Abstract]
19. Cluster [Title/Abstract]
20. OR / 25 – 43

((Cohort stud*) OR (Cohort studies [MeSH]) OR (Cross-sectional studies [MeSH]) OR (Epidemiologic stud*) OR (Epidemiologic studies [MeSH]) OR (Longitudinal stud*) OR (Observational stud*) OR (Population stud*) OR (Prospective stud*) OR (Prospective studies [MeSH]) OR (Retrospective stud*) OR (Retrospective studies [MeSH]) OR (randomized controlled trial [Publication Type]) OR (controlled clinical trial [Publication Type]) OR (randomized [Title/Abstract]) OR (randomly [Title/Abstract]) OR (trial [Title/Abstract]) OR (groups [Title/Abstract]) OR (cluster [Title/Abstract])): 7,571,172 results.

Combined search:

45. #12 AND #24 AND #44

((Short* OR Restrict* OR Reduc* OR Limit*) AND ("Duty hours" OR "Working time" OR "Working hours" OR "Work schedule" OR shift* OR Workload [MeSH])) AND (hospital* OR (Medical Staff, Hospital [MeSH])) AND ((Cohort stud*) OR (Cohort studies [MeSH]) OR (Cross-sectional studies [MeSH]) OR (Epidemiologic stud*) OR (Epidemiologic studies [MeSH]) OR (Longitudinal stud*) OR (Observational stud*) OR (Population stud*) OR (Prospective stud*) OR (Prospective studies [MeSH]) OR (Retrospective stud*) OR (Retrospective studies [MeSH]) OR (randomized controlled trial [Publication Type]) OR (controlled clinical trial [Publication Type]) OR (randomized [Title/Abstract]) OR (randomly [Title/Abstract]) OR (trial [Title/Abstract]) OR (groups [Title/Abstract]) OR (cluster [Title/Abstract]) AND (Internship and residency [MeSH]) OR residen* OR Intern* OR Train* OR Fellow* OR Intern* OR (Medical resident*) OR (Resident physician*))

24/12/2023: 8,357 results
