# Supplementary material for: The impact of shortening shifts of physicians during their residency on patients and physicians: A systematic review and meta-analysis
Source: Isr J Health Policy Res. 2025 Sep 3;14:53. doi: 10.1186/s13584-025-00715-2 (PMC12406601; doi:10.1186/s13584-025-00715-2)

Supplementary Figure 1: Forest plot for 30-day mortality stratified by publication period (before 2010, 2010–2014, 2015–2019).


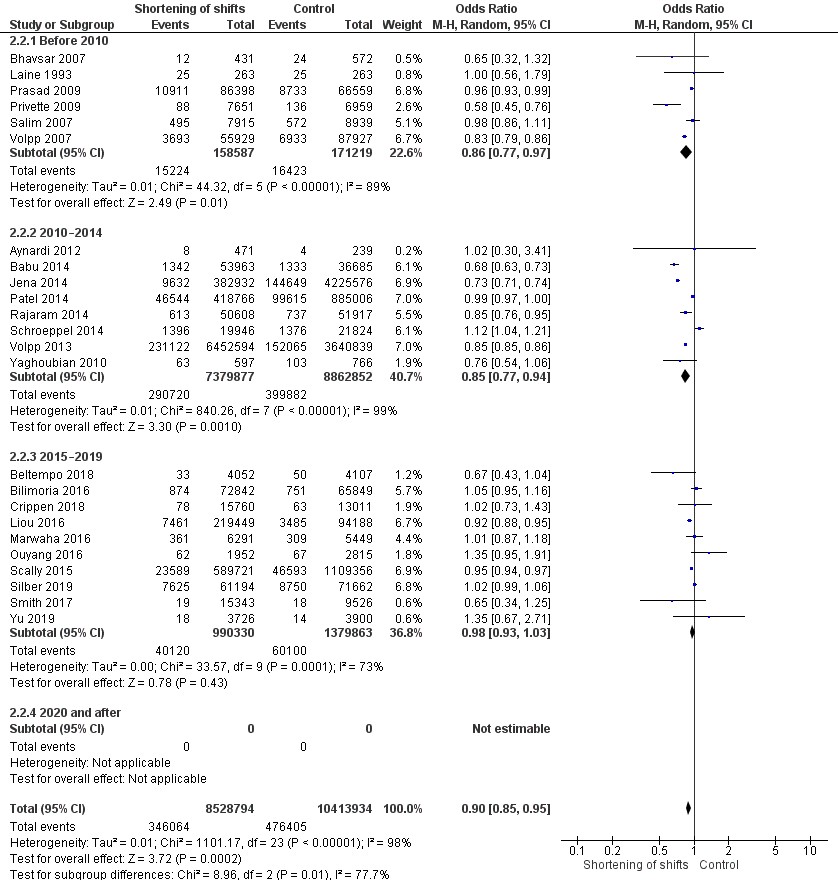


Supplementary Figure 2: Forest plot describing the association of shortening of shifts to 16 hours or less and patient 30-day mortality stratified by publication period (2010–2014 vs. 2015–2019).


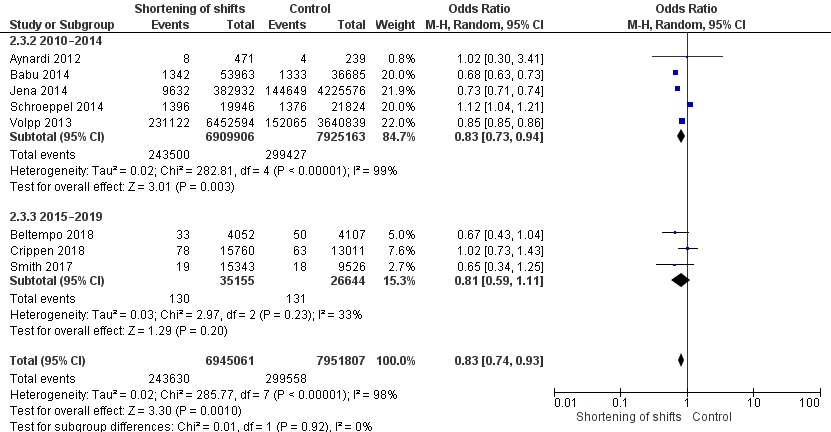


Supplementary Figure 3. Forest plot describing the association of shift shortening and non-surgical complications and adverse events stratified by publication period (before 2010, 2010–2014, 2015–2019, 2020 and after).


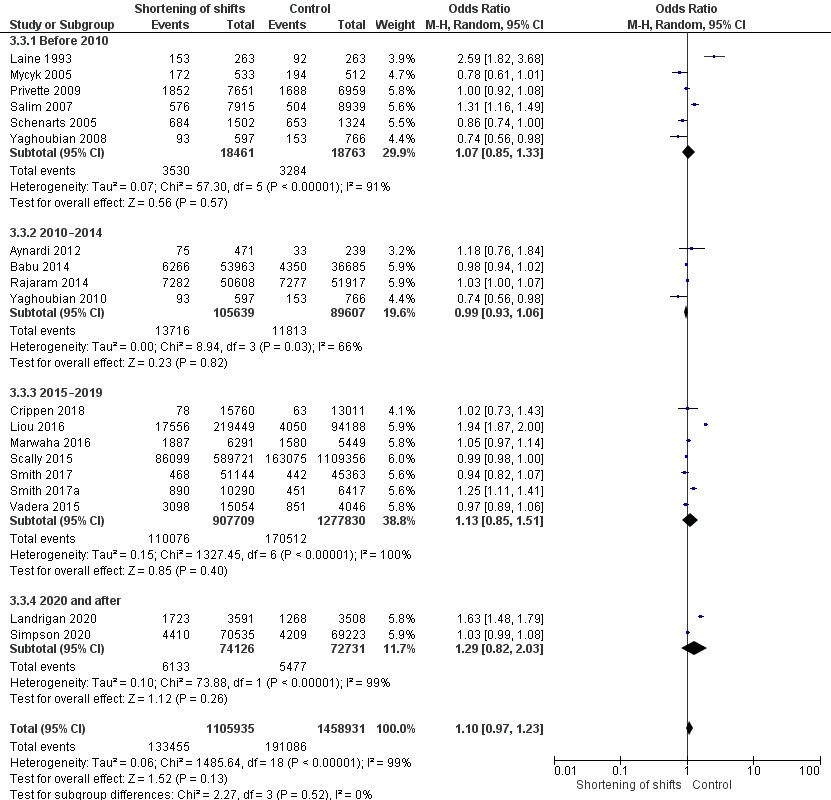


Supplementary Figure 4. Forest plot describing the association of shortening of shifts and operative experience in general surgery residents (measured as mean number of operations per resident) stratified by publication period (before 2011 vs. 2011 and after).


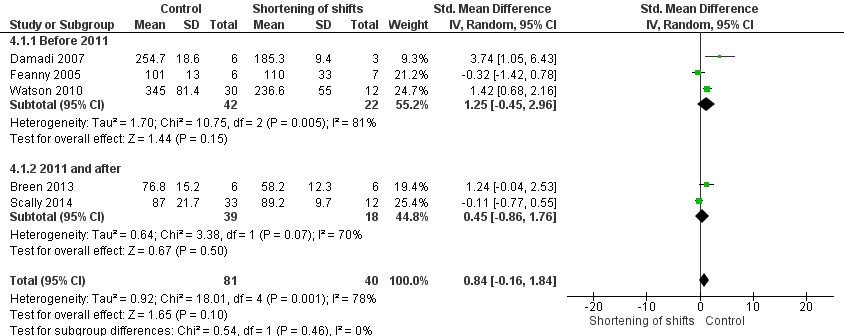

Supplement: Supplementary file 2 — Supplementary Material 2 [file 13584_2025_715_MOESM2_ESM.docx]
