# Supplementary material for: The impact of shortening shifts of physicians during their residency on patients and physicians: A systematic review and meta-analysis
Source: Isr J Health Policy Res. 2025 Sep 3;14:53. doi: 10.1186/s13584-025-00715-2 (PMC12406601; doi:10.1186/s13584-025-00715-2)
Supplement: Supplementary file 3 — Supplementary Material 3 [file 13584_2025_715_MOESM3_ESM.docx]

Supplementary Table 1: Study characteristics

| Study ID | Country | Study Design | Sample size | Population and setting | Intervention type | Key conclusions of study authors |
| --- | --- | --- | --- | --- | --- | --- |
| Alsohime 2021 [126] | Saudi Arabia | Survey | 42 residents | Pediatric residents from two hospitals | Accreditation Council for Graduate Medical Education 2011 (Night float) | The perception of senior residents toward the 24-h on-call system pertains to negative impacts on well-being, education, and patient safety compared with on-call systems with restrictive duty hours, such as the NF system, which is perceived to be less harmful, to exert positive impacts on the quality of delivered healthcare services, and more useful from pedagogic aspect. |
| Amabile 2021 [79] | USA | Retrospective or cross-sectional study | 105,137 procedures | Cardiac surgery patients at 38 hospitals | Accreditation Council for Graduate Medical Education 2011 | The introduction of the 2011 trainees' duty-hour regulations was not associated with worse short-term outcomes for CABG and valve surgery performed in the State of NY by teaching hospitals. |
| Anderson 2017 [63] | USA | Cohort study (concomitant or with historical controls) | 383 | Surgical patients from a single hospital | Accreditation Council for Graduate Medical Education 2011 | We find no correlation between shift length and type of errors committed at our hospital. More flexible work hours did not result in decreases in errors due to handoffs, although longer shifts also did not result in errors due to fatigue. Although there are limitations to this study, we seek to more directly identify types of errors as a result of shift length, rather than only focus on changes in complication rates, as done by the FIRST trial. |
| Arora 2015 [71] | USA | Cohort study (concomitant or with historical controls) | 22,750 patients | Patients admitted to a general medicine teaching service | Both 2003 and 2011 Accreditation Council for Graduate Medical Education | After successive residency duty hours limits, hospitalized patients were more likely to report the attending physician and less likely to report the resident or intern as most involved in their hospital care. Given the importance of experiential learning to the formation of clinical judgment for independent practice, further study on the implications of these trends for resident education and patient safety is warranted. |
| Auger 2014 [101] | USA | Cohort study (concomitant or with historical controls) | 49 residents | Pediatric residents form a single hospital | Accreditation Council for Graduate Medical Education 2003 | Despite the significant decrease in intervention resident presentations on rounds, we did not find differences in the frequency of having at least some contact with faculty or in perceived learning from admissions.  The evening presence of faculty (who were present in both the intervention and control periods) may have blunted the impact of the rounds changes. Regardless, the unavoidable shift in resident learning away from morning rounds, a time when a significant amount of inpatient learning has traditionally occurred, should  encourage resident educators to  capitalize on learning opportunities  throughout the workday. |
| Aynardi 2012 [39] | USA | Cohort study (concomitant or with historical controls) | 722 patients | Orthopedic patients at a large academic institution | Accreditation Council for Graduate Medical Education 2003 | This study found that care of orthopedic patients has not changed despite persistent fears about the 80-hour workweek restrictions or resident turnover months with respect to delay of surgery, complications, and mortality. Tertiary care centers may be seeing less healthy patients; despite this, care appears consistent. This success can be attributed to ancillary staff support, physician extenders, and well-designed patient care protocols. Successful patient outcomes are multifactorial in nature, and a controlled, prospective study may help elucidate the precise reasons for the positive outcomes found in this study. |
| Babu 2014 [37] | USA | Cohort study (concomitant or with historical controls) | 90,648 | Patients undergoing brain tumor and cerebrovascular procedures from a single hospital | Accreditation Council for Graduate Medical Education 2003 | The implementation of duty-hour restrictions correlated with an increased risk of postoperative complications for patients undergoing brain tumor and cerebrovascular neurosurgical procedures. Duty-hour reform may therefore be associated with worse patient outcomes, contrary to its intended purpose. Due to the critical condition of many neurosurgical patients, this patient population is most sensitive and likely to be negatively affected by proposed future increased restrictions. |
| Bailit 2005 [118] | USA | Survey | 28 residents | Obstetrics and gynecology residents form a single hospital | Accreditation Council for Graduate Medical Education 2003 | ACGME reforms altered working conditions for OBGYN residents, but CREOG scores and residents satisfaction with work did not change. ACGME did seem to improve residents satisfaction with their personal lives. |
| Bailit 2004 [66] | USA | Retrospective or cross-sectional study | No information | Obstetrics and gynecology patients from a single hospital | Accreditation Council for Graduate Medical Education 2003 | Although problems in physician performance may be underreported, resident work-hour restrictions show minimal evidence of improvement in quality of care. |
| Barden 2002 [115] | USA | Retrospective or cross-sectional study | 40 residents | General surgery residents form a single hospital | Addition of a night float resident at the junior and PGY-3 levels from 7 PM to 7AM Sunday through Thursday. Other changes included the decrease of ICU call (surgical/trauma, cardiothoracic, and burn ICU) frequency from every 2 to every 3 days. Creation of weekend cross-coverage for the general surgical and vascular services provided residents with two 48-hour “weekends” off per month. | Reduction in resident work hours has salutary effects on basic education for surgical residents. Further research is needed to assess the long-term effects of changes on both residents and patients. |
| Baskies 2008 [96] | USA | Retrospective or cross-sectional study | 109 residents | Orthopedic surgery residents from a large university-based orthopedic surgery residency training program in an urban setting | Accreditation Council for Graduate Medical Education 2003 | To date, concerns over the potential adverse effects of the resident work-hour policies on operative volume for orthopedic surgery residents appear unfounded on the basis of the findings of our study as well as others. Although the objective of this study was to quantify the differences in operative case volumes, it would be important to analyze the quality of the operative experience following the implementation of the work-hour restrictions. This would require an analysis of the individual components of the residency-training program to determine whether there are differences in the types of surgery performed by residents at each level with respect to the implementation of the work-hour restrictions. |
| Beltempo 2018 [27] | USA | Cohort study (concomitant or with historical controls) | 8,159 patients; 185 residents | Infants admitted to a neonatal intensive care unit | Accreditation Council for Graduate Medical Education 2011 | Resident duty hour restrictions were associated with a reduction in the number of yearly hours worked by residents in the NICU as well as a significant decrease in adjusted odds of early mortality but not of hospital mortality in admitted neonates. |
| Bhavsar 2007 [44] | USA | Cohort study (concomitant or with historical controls) | 1003 patients | Cardiological patients hospitalized due to acute coronary syndrome in a single hospital | Accreditation Council for Graduate Medical Education 2003 | Implementation of the Accreditation Council for Graduate Medical Education residency duty-hours restrictions on an academic inpatient cardiology service was associated with improved quality of care and efficiency in patients admitted with acute coronary syndrome. In addition, improved efficiency did not adversely impact patient outcomes, including mortality. |
| Blanchard 2004 [81] | USA | Cohort study (concomitant or with historical controls) | 10 residents | Obstetrics and gynecology residents from an urban, 750-bed tertiary care referral center, serving a significant indigent population, as it is also the county hospital and an urban, 1000-bed tertiary care referral center, serving a largely private local population. Both are teaching hospitals. | Accreditation Council for Graduate Medical Education 2003 | Work hour restrictions have had impact on resident case experience in obstetrics and gynecology. Variance in institutional case numbers account for only some of these changes. |
| Blencowe 2011 [80] | United Kingdom | Cohort study (concomitant or with historical controls) | 12,722 procedures | General surgery residents from a teaching hospital | European working time directive 1993 | The proportion of operating performed by SpRs and SHOs has fallen over the last decade, coinciding with implementation of structural changes to training, the advent of minimally invasive techniques, and the drive for a consultant led health service. Trainees may therefore require increased supervision as well as protected theatre sessions to balance operative training with ward-based duties. Education must be integrated into working practice in order for trainees to achieve expected competencies and ultimately produce adequately experienced consultants. |
| Breen 2013 [74] | Ireland | Retrospective or cross-sectional study | 810 procedures | General surgery residents from a single hospital | European working time directive 1993 | The present data set demonstrates a significant reduction in operative experience gained by SSHOs after local implementation of the EWTD. A major challenge facing Irish surgical training over the next decade is reduced operative exposure in the clinical setting. |
| Chung 2004 [117] | USA | Survey | 2 residents per year | Pediatric residents from a 250-bed community hospital with a level II trauma center | Accreditation Council for Graduate Medical Education 2003 | Reducing work hours cannot be accomplished without reducing educational components. Unlike junior residents, senior residents felt less fulfilled with the new system and do not benefit in physical fatigue. |
| Churnin 2016 [53] | USA | Cohort study (concomitant or with historical controls) | 4,802,979 patients | Neurological patients from a single hospital | Accreditation Council for Graduate Medical Education 2003 | Teaching and nonteaching hospital mortality was similar in patients with nervous system pathology prior to the duty hour reform. While nonteaching institutions demonstrated steadily declining mortality over the decade, teaching hospital mortality spiked in 2004 and declined at a more restricted rate. The timing of these changes could suggest a negative correlation of duty hour restrictions on outcomes of patients with nervous system pathology. |
| Condren 2015 [97] | USA | Retrospective or cross-sectional study | 284 residents | General surgery residents from a tertiary-care academic center | Accreditation Council for Graduate Medical Education 2011 | The duty-hour regulations did not negatively affect objective measures of surgical training in our program. Compliance with the Accreditation Council for Graduate Medical Education duty-hour regulations correlated with an increase in case volume. Adaptations made by the institution, such as maximizing daytime duty hours and increasing physician extenders, likely contributed to the findings. |
| Crippen 2018 [26] | USA | Retrospective or cross-sectional study | 67,158 patients | Patients who underwent otolaryngology surgery nationwide | Accreditation Council for Graduate Medical Education 2003 | In the years following DHR, rates of medical complications, surgical complications, and mortality have significantly improved at non-teaching hospitals. At teaching hospitals, there has been a lack of similar improvement in surgical complications, even after accounting for increasing case volume and complexity in more recent years. While the cause is likely multifactorial, DHR in otolaryngology residency may play a role. |
| Damadi 2007 [76] | USA | Retrospective or cross-sectional study | 6,196 procedures | General surgery residents from two major teaching hospitals, one of which is a verified level 1 trauma center | Accreditation Council for Graduate Medical Education 2003 | Our data suggest that restriction of resident duty hours is associated with a significant decrease in operative experience. |
| Damari 2021 [127] | Israel | Cohort study (concomitant or with historical controls) | 67 residents | Pediatric residents from two hospitals | Shortning of shifts to 13-hours compared to 26 hours | A positive perception was shown regarding the shorter shifts model among Israeli pediatric residents, with a general satisfaction and improved perception of general wellness, ability to deliver quality healthcare, and medical education experience. Given the general change in the workforce worldwide, and changes in priorities of current residents, with a strong emphasis on worklife balance, it is imperative to rethink the traditional way doctors were trained and consider personal wellness and satisfaction when planning residency curriculums. Further research including larger groups and different residency programs during non-pandemic times and longer periods of follow-up are needed. |
| DeLaroche 2013 [124] | USA | Survey | 825 residents | Pediatric residents nationwide | Accreditation Council for Graduate Medical Education 2011 | This national survey demonstrates that the decrease in duty hours mandated by the ACGME in 2011 was achieved without significantly impacting pediatric interns’ overall neonatal experience, knowledge or self assessed confidence. |
| Durkin 2008 [89] | USA | Cohort study (concomitant or with historical controls) | No information | General surgery residents from a single hospital | Accreditation Council for Graduate Medical Education 2003 | ABSITE scores improved significantly after the restriction of resident work hours. Resident operative experience was not affected. An unexpected consequence of work-hour restrictions may be an improvement in surgical resident education. |
| Elbadrawy 2008 [82] | United Kingdom | Retrospective or cross-sectional study | 16 residents | Obstetrics and gynecology residents from a a teaching district hospital | European working time directive 1993 | Gynecology surgical activity in the Singleton hospital has changed significantly over the last decade. There has been a reduction in training opportunities in gynecological surgery after the implementation of the Calman reforms and EWTD. The reduction in cases performed by trainees is likely to increase the risk of producing future consultants with limited gynecological surgical experience. |
| Feanny 2005 [77] | USA | Retrospective or cross-sectional study | 13 residents | General surgery residents from a 700-bed city hospital and level 1 trauma center | Accreditation Council for Graduate Medical Education 2003 | The ACGME regulatory environment is adversely affecting the emergency operative experience of surgical residents. Our findings underscore the need to develop alternative methods to augment the residents' operative experience. |
| Ferguson 2005 [83] | USA | Retrospective or cross-sectional study | 11,835 procedures | General surgery residents from a single hospital | Accreditation Council for Graduate Medical Education 2003 | Work-hour limitation can be devised to maximize resident education, optimize patient care, and maintain resident operative volume. Although some changes seemed to increase the operative case volume for PGY5 residents, others had no effect. There does not seem to be a clear relationship between types of changes and case volume. At the PGY1 level, Q4 and PE changes decreased operative experience on 1 rotation but not on another, although the difference in this decrease seems clinically insignificant. Individualization of changes to meet the needs of specific rotations seems more important than specific changes in coverage pattern. Perhaps the most important finding is that changes can be made to bring work hours into compliance without materially effecting operative case volume. |
| Gelfand 2004 [116] | USA | Survey | 37 residents | General surgery residents from four urban, university-based centers | Accreditation Council for Graduate Medical Education 2003 | Despite successful reductions in resident work hours, measures of burnout were not significantly affected. However, important clinical activities such as time spent in the operating room, clinic, and making rounds were maintained. Formal in-hospital education time was reduced. |
| Gopaldas 2009 [43] | USA | Cohort study (concomitant or with historical controls) | 1562 patients | Cardiac surgery patients in Veterans Affairs hospitals | Accreditation Council for Graduate Medical Education 2003 | Cardiac operations performed after the resident work-hour restriction went into effect were associated with significantly lower adjusted 30-d and 6-mo mortality rates than were operations performed before the work-hour restriction became effective. |
| Hanlon 2008 [109] | Canada | Survey | 308 residents | Family medicine residents nationwide | Accreditation Council for Graduate Medical Education 2011 | Many anesthesia residents do exhibit excessive daytime sleepiness, with a similar incidence for those working within either modified or traditional call systems. Our study suggests that sleepiness may be reduced by scheduling on-call duties no more frequently than one in every five nights and by ensuring that residents sleep more than 2 h while on call. |
| Hopmans 2015 [91] | The Netherlands | Retrospective or cross-sectional study | 235,357 procedures | General surgery residents from 1 university hospital and 6 district training hospitals | European working time directive 1993 | Implementation of the European Working Time Directive has not affected adversely the number of surgical procedures performed by residents within a general surgical training region in the Netherlands. |
| Hutter 2006 [119] | USA | Cohort study (concomitant or with historical controls) | 116 residents | Mixed Specialties | Accreditation Council for Graduate Medical Education 2003 | The data presented demonstrate many concerns with regards to the professional development of future surgeons, including a change toward a shift-worker mentality that is not patient-focused, less continuity of care with loss of critical information with each handoff, and a decrease in the patient/doctor relationship. Although the mandated restriction of resident work-hours has had no measurable effect on the quality of patient care, there have been significant improvements in the current quality of life of residents. |
| Jagsi 2006 [120] | USA | Survey | 1770 residents | Residents with mixed specialties from two hospitals | Accreditation Council for Graduate Medical Education 2003 | This study shows that it may be possible to reduce residents' hours--and the perceived adverse impact of fatigue--while generally preserving the self-assessed quality, quantity, and outcomes of graduate medical education. |
| Jagsi 2008 [134] | USA | Survey | 1770 residents | Residents with mixed specialties from two hospitals | Accreditation Council for Graduate Medical Education 2003 | It is possible to reduce residents’ hours without increasing patient load. Doing so may reduce the extent to which fatigue affects patient safety as perceived by these frontline providers. |
| Jaradat 2020 [128] | Jordan | Survey | 180 residents | Mixed Specialties | Accreditation Council for Graduate Medical Education 2003 | Poor sleep quality, depression, and anxiety remain highly prevalent among resident physicians. This study shows that these rates can be reduced by optimizing shift scheduling and duration, which may positively affect the efficiency of their training and continuity of patient care. We also recommend that public health authorities specifically tailor sleep hygiene practices to address residents’ needs and increase the medical workforce to better enhance sleep quality and the psychological and physical well-being of resident doctors. |
| Jarman 2004 [86] | USA | Cohort study (concomitant or with historical controls) | 325 residents | General surgery residents from a single hospital | Accreditation Council for Graduate Medical Education 2003 | Work-hour restrictions result in a significant decrease in operative experience. This detriment can be partially alleviated with the institution of a night rotation to better regulate in-house call. |
| Jena 2014 [35] | USA | Cohort study (concomitant or with historical controls) | 4,608,508 patients | Patients admitted in acute care hospitals | Both 2003 and 2011 Accreditation Council for Graduate Medical Education | In summary, our study suggests that the 2003 ACGME duty hour reforms did not adversely impact hospital mortality and length of stay of patients cared for by new attending physicians who were partly or fully exposed to reduced duty hours during their own residency. Further assessment of the impact of the 2003 and 2011 duty-hour reforms on other aspects of physician quality, for particular patient sub-populations, and in national data is important. |
| Johnson 2018 [102] | USA | Retrospective or cross-sectional study | No information | Orthopedic surgery residents nationwide | Accreditation Council for Graduate Medical Education 2011 | There has been little data to support the theory that resident work hour restrictions have improved education or patient care in any meaningful way. In this study, there was a statistically significant increase in publications after 2011; however, the number of publications between NIH funded and non-NIH funded programs did not differ. This study is the first to demonstrate that with increasing duty hour restrictions, orthopedic surgery residents may be using more of their free time to conduct research. |
| Kane 2021[65] | Ireland | Retrospective or cross-sectional study | 18,761 patients | Obstetrics and gynecology patients from a large tertiary referral university hospital | European working time directive 1993 | This study shows an association between obstetric trainee working practices, RG1 CS and OVD rates; this is most pronounced at night and after the introduction of the EWTD. It is unlikely that obstetric trainee working practices are the only factor related to the increasing CS rate and reduced OVD rate. Consideration should be giving to addressing the needs of obstetric trainees in relation to achieving their competencies with now reduced labor ward exposure. Further study is required to see if alternate arrangements in relation to simulation training could increase the OVD rate and reduce the CS rate. |
| Kashner 2010 [121] | USA | Retrospective or cross-sectional study | 19,605 residents | Mixed Specialties | Accreditation Council for Graduate Medical Education 2003 | The 2003 ACGME duty hours standards were associated with improved satisfaction for resident clinical training and learning environments. |
| Krug 2017 [125] | USA | Survey | 345 residents | Internal medicine residents from a single hospital | Both 2003 and 2011 Accreditation Council for Graduate Medical Education | Validated measures of resident wellbeing changed across the three time points measured. Residents had the lowest rates of burnout and depression in 2012. Resident perceptions of the 2011 WHLs, however, were generally negative. |
| Laine 1993 [46] | USA | Cohort study (concomitant or with historical controls) | 526 patients | Internal medicine patients | Accreditation Council for Graduate Medical Education 2003 | These results suggest that restricted house staff working hours were associated with delayed test ordering by house staff and increased in-hospital complications. While these potentially deleterious effects on the quality of care did not result in statistically significant differences in more serious outcomes, further study at other hospitals is warranted to determine staffing strategies that optimize quality of care for patients, as well as medical education and quality of life for house officers. |
| Landrigan 2008 [135] | USA | Cohort study (concomitant or with historical controls) | 268 residents | Pediatric residents from two hospitals | Accreditation Council for Graduate Medical Education 2003 | Total hours of work and sleep did not change after implementation of the duty hour standards. Although fewer residents were burned out, rates of medication errors, resident depression, and resident injuries and educational ratings did not improve. |
| Lee 2003 [62] | Hong Kong | Cohort study (concomitant or with historical controls) | 16 residents | Mixed Specialties | Accreditation Council for Graduate Medical Education 2003 | We conclude that the night shift call system can reduce the number of consecutive hours worked, and is associated with significantly less impaired cognition in basic surgical trainees. In hospitals with small training programs like ours, extra relieving staffs are required to provide daytime coverage. |
| Lim 2006 [90] | United Kingdom | Cohort study (concomitant or with historical controls) | 3312 procedures | Cardiac surgery residents from a single hospital | Accreditation Council for Graduate Medical Education 2003 | With a successful institution specific training module and a commitment to training, sustained exposure to operative surgical training can be achieved despite shortening working hours. Close surveillance and further audit is essential to determine if the improvements to surgical training can be sustained with the anticipated decline in the workload for coronary surgery. |
| Lindbloom 2014 [104] | USA | Retrospective or cross-sectional study | No information | Family medicine residents from a single hospital | Accreditation Council for Graduate Medical Education 2011 | New duty hour regulations not only limit the time resident physicians spend in the hospital but also their experience in the ambulatory setting. Considering the emphasis family medicine training programs place on continuity of care and the PCMH, the new regulations will have significant implications for these programs. |
| Liou 2016 [31] | USA | Cohort study (concomitant or with historical controls) | 3312 procedures | Surgical trauma patients nationwide | Accreditation Council for Graduate Medical Education 2003 | Although there may be some benefit to resident duty hour restrictions, there is still room for improvement in patient care. Individual institutions should carefully review their own complication data to identify preventable systems issues, such as poor handoffs, and opportunities for increased resident supervision. |
| Liu 2018 [133] | Canada | Cohort study (concomitant or with historical controls) | 313,637 patients | Obstetrical patients who underwent an overnight delivery of any kind between midnight and 7:00 AM in a single hospital | Accreditation Council for Graduate Medical Education 2011 | We found that the implementation of the night float system for Obstetrical residents in Toronto did not cause a change in overall patient outcomes, but there was an increased incidence of transfusion and PPH and surgical/obstetrical complications. These results suggest that duty hour restrictions may not be as beneficial as initially thought, and adds to the controversial data from other specialties. It certainly highlights the need to further investigate the clinical impact a change in resident duty hours has, as well as the effect on resident well-being and education, to determine the best strategy to adopt moving forward. |
| Lockley 2004 [110] | USA | Cohort study (concomitant or with historical controls) | 11947 patients | Internal medicine from a single hospital | Accreditation Council for Graduate Medical Education 2011 | Eliminating interns’ extended work shifts in an intensive care unit significantly increased sleep and decreased attentional failures during night work hours. |
| Mahesh 2014 [92] | United Kingdom | Cohort study (concomitant or with historical controls) | 6688 procedures | Cardiac surgery residents from a single high-volume [>1500 procedures/year] adult cardiac surgical center | European working time directive 1993 | Implementation of the final phase of EWTD has not decreased training in a high-volume center. The positive adjustment of trainers' attitudes and efforts to match trainees' needs allow maintenance of adequate training, despite reduction in working hours and increasing patients' risk profile. |
| Markelov 2011 [84] | USA | Retrospective or cross-sectional study |  | General surgery residents from a community hospital | Accreditation Council for Graduate Medical Education 2003 | Comparing the national trend to the community hospital we see that there is total increase in cases at the national level whereas there is a decrease in case volume at the community hospital. These trends can also be followed in ACGME defined subcategories which form the major case load for a general surgical training such as alimentary tract, abdominal, breast, and vascular procedures. We hypothesize that work hour restrictions have been favorable for the larger programs, as these programs were able to better integrate the night float system, restructure their call schedule, and implement institutional modifications which are too resource demanding for smaller training programs. |
| Marwaha 2016 [30] | USA | Retrospective or cross-sectional study | 11,740 Patients | Patients admitted to a hospital- trauma service | Accreditation Council for Graduate Medical Education 2011 | Although most major indicators of morbidity and mortality remained insulated from change after the implementation of the 2011 duty hour reform, many secondary measures of quality in trauma care still changed. In particular, practice patterns related to resource use, such as the use of bedside procedures and the number of OR visits, increased most consistently. No secondary variables exhibited improvements strongly associated with the reform. Changes in these measures were not accurately reflected in the behavior of major outcomes and, in fact, suggest that less-commonly studied areas of quality in the context of the 2011 duty hour reform, such as cost of care, should be studied. These institutional trends should be validated on a national level. |
| Maxwell 2010 [72] | United Kingdom | Retrospective or cross-sectional study | 200 episodes | Neurosurgery residents from a regional neurosurgical unit | European working time directive 1993 | The EWTD has had a direct adverse effect on continuity of patient care in neurosurgery. The amount of training time for neurosurgical residents has also been substantially reduced. Without prompt recognition of this problem and appropriate remedial action, there may be a continued decline in standards of neurosurgical patient care and the clinical ability of trained neurosurgeons. |
| McElearney 2005 [93] | USA | Retrospective or cross-sectional study | No Information | Mixed Specialties | Night float 14-hour shifts (6 pm–7:15 am) | PGY5s did have statistically fewer cases after the work-hours restriction, which likely represented shifting of postcall afternoon cases to other residents. Comparing other classes and all PGYs, case numbers were not statistically different. Operative training experience does not appear to be hindered by the 80-hour work week. |
| Morrison 2009 [50] | United States and Puerto Rico | Retrospective or cross-sectional study | 492,173  Patients | Injured patients nationwide | Accreditation Council for Graduate Medical Education 2003 | Despite the great deal of controversy surrounding the 80-h work week, few papers exist that specifically examine patient mortality within the field of trauma surgery. This large retrospective analysis demonstrates slightly decreased mortality and morbidity among trauma patients in university hospitals nationwide after implementation of the 80-h work week, even when controlling for possible confounders. Although these differences are not likely to be clinically important, the data are statistically very significant. Therefore, we conclude that the 80-h work week has not resulted in any significant deterioration in patient outcomes in this particular population. |
| Mycyk 2005 [54] | USA | Retrospective or cross-sectional study | 1,065 Patients | Patients admitted to a 750-bed academic tertiary care hospital | Accreditation Council for Graduate Medical Education 2003 | Hospitalwide ADEs remained constant despite limiting of resident physician weekly work hours to 80. |
| Nomura 2016 [111] | Japan | Cohort study (concomitant or with historical controls) | 41 residents | Pediatric residents from a 490-bed tertiary care pediatric and perinatal hospital | 8 hours off work after the morning round (~22 hour shifts) compared to working for more than 30 consecutive hours | Although duty hour regulations have been used as the first-line solution for the prevention of burnout and depression among residents, this study found no evidence of the effectiveness of this approach. Resident wellness programs represent an additional strategy and should be aimed at fostering peer support and improvement of resident–faculty interactions. Such an approach could create a confidential and mutually beneficial relationship between physicians of different generations with conflicting belief structures. |
| Occhino 2011 [95] | USA | Cohort study (concomitant or with historical controls) | 15 residents | Obstetrics and gynecology residents from two hospitals | Accreditation Council for Graduate Medical Education 2003 | Duty-hour restrictions have not adversely affected the operative experience of obstetrics and gynecology residents. No significant differences in the number of the spontaneous vaginal deliveries, abdominal hysterectomies, or vaginal hysterectomies performed were observed. |
| Ouyang 2016 [29] | USA | Cohort study (concomitant or with historical controls) | 3,450 patients | Internal medicine patients from a single hospital | Accreditation Council for Graduate Medical Education 2011 | Patients taken care of by housestaff working more than 80 hours per week had increased length of stay and number of ICU transfers. There was no association between resident work-hours and patient in-hospital mortality or 30-day readmission rate. |
| Patel 2014 [34] | USA | Retrospective or cross-sectional study | Medical patients 2,790,356  Surgical patients 3,593,917 | Medicare patients admitted to short-term, acute care, general US nonfederal hospitals | Accreditation Council for Graduate Medical Education 2011 | Among Medicare beneficiaries, there were no significant differences in changes in 30-day mortality rates or 30-day all-cause readmission rates for those hospitalized in more intensive relative to less intensive teaching hospitals in the year after implementation of the 2011 ACGME duty hour reforms compared with those hospitalized in the 2 years before implementation. |
| Paul 2012 [94] | UK | Cohort study (concomitant or with historical controls) | 44 residents | Family medicine residents from a single hospital | European working time directive 1993 | Our data contradict the hypothesis that the European Working Time Directive has reduced access to training, or suggest that if it has, other factors (such as improved trainee rostering) have overridden its effect. |
| Pepper 2014 [86] | USA | Cohort study (concomitant or with historical controls) | 656 events | Internal medicine residents from an academic hospital | Accreditation Council for Graduate Medical Education 2011 | Small but significant decrease was found in resident academic performance after implementation of new resident duty hours. We also observed that targeted changes in resident on-call schedules reduce code blue-related transfers to the intensive care unit. Medical emergency teams and rapid response teams with objective activation criteria also may contribute to decreased numbers of code blue events. We need large multicenter studies to corroborate these findings. |
| Poulose 2005 [67] | USA | Retrospective or cross-sectional study | A mean of 2.6 million discharges per year | Surgical patients nationwide | Accreditation Council for Graduate Medical Education 2003 | Resident work hour limits in New York teaching hospitals were not associated with improvements in surgical patient safety measures, with worsening trends observed in APL and PEDVT corresponding with enforcement. |
| Prasad 2009 [42] | USA | Cohort study (concomitant or with historical controls) | 230,151 Patients | Patients admitted to 104 different medical and surgical intensive care units from non-teaching, community with residents, or academic based hospitals | Accreditation Council for Graduate Medical Education 2003 | There was a decrease in in-hospital mortality in ICU patients during the years of observation. This decrease was not associated with hospital teaching status, suggesting no net positive or negative association of the resident work-hours regulations with a major patient centered outcome. |
| Privette 2009 [41] | USA | Cohort study (concomitant or with historical controls) | 14,610 patients | Surgical patients from a single academic institution to the general, vascular, and trauma surgery services | Accreditation Council for Graduate Medical Education 2003 | Implementation of RWHR was associated with reduced provider-related complications and mortality suggesting improved patient safety. This was likely due to several factors including reduced resident fatigue and greater attending involvement in clinical care. |
| Rajaram 2014 [33] | USA | Retrospective or cross-sectional study | 204,641 Patients | General surgery residents nationwide | Accreditation Council for Graduate Medical Education 2003 | Implementation of the 2011 ACGME duty hour reform was not associated with a change in surgical patient outcomes or resident examination performance. The implications of these findings should be considered when evaluating the merit of the 2011 ACGME duty hour reform and revising related policies in the future. |
| Rajaram 2016 [129] | USA | Retrospective or cross-sectional study | 73 teaching and 2043 non-teaching hospitals | Patients admitted in Medicare and Medicaid Service Hospitals | Accreditation Council for Graduate Medical Education 2011 | The 2011 ACGME duty hour reform was not associated with improvements in process-of-care and patient experience measures. These data should be considered when considering reform of resident duty hour policies. |
| Rashid 2012 [64] | Pakistan | Cohort study (concomitant or with historical controls) | 144 patients | Patients presenting to a university Hospital with femoral inter-trochanteric fractures who underwent dynamic Hip screw fixation | Accreditation Council for Graduate Medical Education 2003 | Resident work hour reform was associated with a significant decrease in the mean operative time for patients undergoing DHS fixation. However morbidity and mortality following DHS fixation for Intertrochanteric fractures has not decreased after implementation of these reforms. Further research evaluating patient outcomes in orthopedic surgery following work-hour restrictions are needed. Moreover, the impact of these reforms on the educational and research activities of the residents also needs to be determined. |
| Richter 2014 [123] | Germany | Retrospective or cross-sectional study | 1,376 residents | Mixed Specialties | European working time directive 1993 | Changes in working conditions in accordance with the European Working Time Directive are not accompanied by reduced strain and risk of burnout for physicians. Rather, data argue for greater intensification in work, especially for senior physicians. Further studies are suggested in order to explore interventions for a sustainable improvement in the working conditions of physicians. |
| Ripp 2015 [112] | USA | Survey | 231 residents | Internal medicine residents from two academic medical centers | Accreditation Council for Graduate Medical Education 2011 | Job burnout and self-reported sleepiness in IM resident physicians were unchanged after the 2011 DHRs at three academic institutions. Further investigation into the determinants of burnout can inform effective interventions. |
| Rosenbluth 2013 [130] | USA | Cohort study (concomitant or with historical controls) | Medical patients: 664; Surgical patients: 290 | Pediatric patients from a primary pediatrics medical-surgical unit | Accreditation Council for Graduate Medical Education 2011 | Increased costs related to compliance with new ACGME standards are estimated by the Institute of Medicine to be as high as $1.7 billion. The costs will likely be directed toward additional staffing as many residency programs hire additional trainees and many hospital systems add nocturnists. Although these costs cannot be directly recouped through billing, our findings of reduced LOS and TC suggest that these costs may be partially offset by improved care efficiency. |
| Salgado 2022 [51] | USA | Cohort study (concomitant or with historical controls) | 62 residents | Internal medicine patients from a single hospital | Accreditation Council for Graduate Medical Education 2011 | This prospective, controlled study suggests that objective outcomes of patient care and patients’ perceptions of care are not significantly affected by extended intern duty hours on a general medicine service. However, this study raises concerns about the implementation of extended duty hours given the increased fatigue and significant dissatisfaction among interns and residents as indicated by anonymous survey data. |
| Salim 2007 [49] | USA | Cohort study (concomitant or with historical controls) | 16854 patients | Trauma patients from a single hospital | Accreditation Council for Graduate Medical Education 2003 | Although there was no difference in deaths between the 2 time periods, there was a significant increase in total, preventable, and no preventable complications. This increase in complication rate may be due, in part, to the new 80-hour workweek policy. |
| Sarff 2009 [131] | USA | Cohort study (concomitant or with historical controls) | 36 residents | Internal medicine residents from a single hospital | Accreditation Council for Graduate Medical Education 2003 | Exposure to minor cancer cases and endoscopies has decreased; this has led to a requirement for a minimum number of endoscopies/graduating resident, and to strategies for increasing exposure to minor cancer cases. |
| Scally 2014 [73] | USA | Retrospective or cross-sectional study | 50 residents | General surgery residents from a university hospital | Accreditation Council for Graduate Medical Education 2011 | The call schedule attempts to minimize prolonged night-float coverage responsibilities for interns in hopes of preserving their operative experience. In spite of increased duty hour restrictions, PGY-1 operative volume has not decreased significantly. However, in the same time period, PGY-2 and PGY-3 case volume has increased. The findings highlight the challenges faced by surgical residencies in light of these new restrictions, particularly the 16-hour limit. Additional rigorously designed prospective studies should be conducted to better understand the influence of the most recent Accreditation Council for Graduate Medical Education work hour limitations on the subjective and objective experiences of surgical residents. |
| Scally 2015 [32] | USA | Retrospective or cross-sectional study | 1,699,077 patients | Patients undergoing general and vascular surgery procedures | Accreditation Council for Graduate Medical Education 2011 | In Medicare beneficiaries undergoing surgery at teaching hospitals, outcomes have not improved since the 2011 ACGME duty hour regulations. |
| Schenarts 2005 [55] | USA | Cohort study (concomitant or with historical controls) | 2826 patients | Patients admitted to a university level 1 trauma service | Accreditation Council for Graduate Medical Education 2003 | Resident work-hour restrictions were not associated with significant improvement or deterioration in patient outcome. |
| Schroeppel 2014 [36] | USA | Cohort study (concomitant or with historical controls) | 41,770 patients | Trauma patients from a regional trauma center | Accreditation Council for Graduate Medical Education 2003 | The work hour restrictions implemented by the ACGME in 2003 have changed the way academic medicine is practiced and taught. The theory behind the restrictions was improving patient care, improving ex- amination scores, and increasing resident morale. Many of these factors have yet to materialize in a significant way. In an era of cost containment, increasing LOS with less efficient medicine in academic hospitals is an alarming finding. We expect that in further studies with the 2011 amendment, this will only get worse. |
| Schumacher 2015 [113] | USA | Survey | 1,251 residents | Pediatric residents nationwide | Both 2003 and 2011 Accreditation Council for Graduate Medical Education | Overall, the effects of duty hour limitations on fatigue seem to be positive in this study. However, concerns about adequate training and variable fatigue effects reported with respect to patient errors continue to make this an important area for future research to guide continued understanding for how to best structure the resident working and learning environment for the safety and benefit of all within it. |
| Shelton 2014 [132] | USA | Retrospective or cross-sectional study | Teaching 34 million patients  Non teaching 44 million Patients | Patients treated by residents with mixed specialties nationwide | Accreditation Council for Graduate Medical Education 2003 | Trends in rates for 2 of the 6 PSIs changed significantly after DHR implementation, with PTx rates worsening in T hospitals and PEDVT rates worsening in NT hospitals. Lack of consistent patterns of change suggests no measurable effect of the policy change on these PSIs. |
| Shetty 2007 [52] | USA | Cohort study (concomitant or with historical controls) | medical patients 1,268,738  surgical patients 243,207 | Medical and surgical patients nationwide | Accreditation Council for Graduate Medical Education 2003 | The work-hour regulations were associated with decreased short-term mortality among high-risk medical patients in teaching hospitals but were not associated with statistically significant changes among surgical patients in teaching hospitals. |
| Short 2006 [88] | USA | Cohort study (concomitant or with historical controls) | 35 residents | Obstetrics and gynecology residents from a military major teaching hospital | Accreditation Council for Graduate Medical Education 2003 | The 80-hour workweek restriction resulted in similar total numbers of obstetrics and gynecology cases, although the total number of obstetric cases per resident declined after implementation. |
| Simpson 2020 [60] | USA | Retrospective or cross-sectional study | 28,403 patients | Patients who underwent breast reconstruction surgery nationwide | Accreditation Council for Graduate Medical Education 2003 | This study demonstrates that complication rates after breast reconstruction did not significantly change after implementation of the 2011 duty hour reform. |
| Smith 2008 [105] | Canada | Survey | 12 residents | Pediatric residents from a single hospital | 18 hour on-call shifts compared to 26.5 hour on-call shifts | Reduced mental attention after being on call is more pronounced after longer shifts. Learning was not affected by shift duration or by how recently trainees were on call. Increased patient familiarity does not augment learning in patient-based medical education. |
| Smith 2017 [28] | USA | Retrospective or cross-sectional study | 96,507 procedures | Surgical head and neck patients nationwide | Accreditation Council for Graduate Medical Education 2003 | Overall complication rates did not change for head and neck key indicator procedures. Moreover, concerns about reduced surgical case numbers appear unfounded, especially for otolaryngology programs. |
| Smith 2017a [59] | USA | Retrospective or cross-sectional study | 96,507 procedures | Surgical head and neck patients nationwide | Accreditation Council for Graduate Medical Education 2003 | While recurrent laryngeal nerve injury, hematoma formation, and hypoparathyroidism did not change, length of stay and mortality improved within THs-OTO following head and neck endocrine procedures after implementation of duty hour regulations. This finding refutes the concern that duty hour restrictions result in poorer overall outcomes. Less time available to develop technical competence may play a factor in some outcomes in lieu of recurrent laryngeal nerve injury increasing within THs and accidental injury to vessels, organs, or nerves and hypocalcemia increasing within THs-OTO. Furthermore, head and neck endocrine cases increased at THs with otolaryngology programs. |
| Spencer 2005 [78] | USA | Retrospective or cross-sectional study | 47 residents | Pediatric residents from an academic medical center | Accreditation Council for Graduate Medical Education 2003 | The 80-hour limit has had minimal impact on residents’ operative experience, in case number and variety, and residents’ perceptions of their educational experience. Residents’ reduction in duty hours may have been achieved at the expense of outpatient clinic experiences. |
| Stienen 2019 [87] | Switzerland | Survey | 80 residents | Neurosurgery residents nationwide | European working time directive 1993 | The preliminary analysis of the first 80 responses now provides a first reference frame for caseload that can be used by current and future European residents to critically compare their own operative numbers to. There was a strong decline in surgical cases over time, and trainees graduating after introduction of the European WTD 2003/88/EC had less surgical exposure. |
| Vadera 2015 [57] | USA | Retrospective or cross-sectional study | 27,941,417 patient admissions | General surgery patients nationwide | Accreditation Council for Graduate Medical Education 2003 | After ACGME duty-hour reform, medication error rates increased in teaching hospitals, which diminished over time. This decrease in errors may be related to changes in training program structure to accommodate duty-hour reform. |
| Volpp 2007 [45] | USA | Retrospective or cross-sectional study | 318,636 patients | Patients admitted to acute-care Veterans Affairs (VA) hospitals with diagnoses of acute myocardial infarction (AMI), congestive heart failure, gastrointestinal bleeding, or stroke or a diagnosis related group classification of general, orthopedic, or vascular surgery. | Accreditation Council for Graduate Medical Education 2003 | We found that the duty hour reforms were associated with a significant improvement in mortality in more teaching-intensive VA hospitals for patients with medical conditions. Furthermore, we did not find an increase in mortality associated with the new rules affecting surgical patients. Further assessment of how the reforms affected other clinical and educational outcomes in both VA and non-VA settings would be important before modification of the current duty hour standards. |
| Volpp 2013 [38] | USA | Retrospective or cross-sectional study | 13,678,956 patients | Medicare patients admitted to short-term acute care non-federal hospitals with principal diagnoses of acute myocardial infarction (AMI), gastrointestinal bleeding, or congestive heart failure (CHF); or a diagnosis-related group (DRG) classification of general, orthopedic, or vascular surgery. | Accreditation Council for Graduate Medical Education 2003 | Duty hour reform was associated with no significant change in mortality in the early years after implementation, and with a trend toward improved mortality among medical patients in the fourth and fifth years. It is unclear whether improvements in outcomes long after implementation can be attributed to the reform, but concerns about worsening outcomes seem unfounded. |
| Vucicevic 2014 [99] | USA | Retrospective or cross-sectional study | 171 residents | Internal medicine residents from a single hospital | Accreditation Council for Graduate Medical Education 2011 | The implementation of the 2011 ACGME duty hour regulations resulted in a decrease in the numbers of patients seen by residents in the in-patient setting; with a strong trend to fewer patients admitted each day. There was no change in the outpatient ACGME duty hour restrictions exposure and test scores and an increase in conference attendance was noted. Whether these findings will translate into differences in patient outcomes or quality of care will require longer term studies. |
| Watson 2010 [75] | USA | Retrospective or cross-sectional study | 40 residents | General surgery residents from a single hospital | Accreditation Council for Graduate Medical Education 2003 | Despite an increase in the total number of major operative cases available, the volume of cases performed by residents has decreased after implementation of the Accreditation Council for Graduate Medical Education (ACGME) work-hour restrictions. Our data suggest that the impact of the 80-hour workweek has had a detrimental effect on the conventional resident training experience. |
| Weaver 2020 [114] | USA | Cohort study (concomitant or with historical controls) | 21862 residents | Mixed Specialties | Accreditation Council for Graduate Medical Education 2011 | The 2011 ACGME guidelines that reduced work hours of first-year resident physicians was associated with improved resident safety and health. Extended-duration shifts and prolonged weekly work hours continue to adversely impact the safety and well-being of resident physicians. There is a pressing need to monitor changes in the rates of these adverse outcomes now that extended-duration shifts have been re-introduced. |
| Yaghoubian 2008 [58] | USA | Retrospective or cross-sectional study | 2470 patients | Post laparoscopic cholecystectomy patients | Accreditation Council for Graduate Medical Education 2003 | At a major public teaching hospital, the bile duct injury rate and the overall complication rate decreased after implementation of the 80-hour workweek. |
| Yaghoubian 2010 [40] | USA | Retrospective or cross-sectional study | 1432 patients | Trauma patients who required an urgent/emergent operation by the a trauma surgery service at a Level 1 trauma center | Accreditation Council for Graduate Medical Education 2011 | Trauma surgery performed at night by residents who have worked longer than 16 hours have similar favorable outcomes compared with those performed during the day. Instituting a 5-hour rest period at night is unlikely to improve outcomes of these commonly performed operations |
| Yu 2019 [25] | Korea | Retrospective or cross-sectional study | 7,626 patients | Mixed Specialties | Night float system, only 70% of the residents worked on day duty, while the remaining 30% of residents worked on night duty. Residents on night duty worked for 12 hours at night for 5 days a week (on weekdays only) compared to 36 hours of continuous work | Resident working hour restrictions can be matched systematically without compromising patient safety. On the contrary, the NF system showed beneficial outcomes in terms of reduced postoperative bleeding for ward patients and faster response to patients in the emergency room leading to prompt management. Associated medical staff were also more satisfied with the NF system and considered this system to be beneficial overall for the hospital. |
| Zahrai 2011 [122] | Canada | Survey | 16 residents | Orthopedic surgery residents from two level-1 trauma centers | Night float 14-hour shifts (5 pm–7 am) compared to 24 hours shift. | The residents in the standard call group had better health-related quality of life compared with those in the night float group. No differences existed in subjective educational benefits and stress level between the groups. |
| Barger 2019 [103] Landrigan 2020 [56]  Rahman 2021 [1] | USA | Cluster-randomized, crossover, non-inferiority trial- ROSTERS | 312 residents (358 resident-physician rotations) | Pediatric intensive care unit residents from 6 hospitals | Up to 16 consecutive hour shifts (RCWR) compared to standard 24-28 hour shifts (EDWR) | Barger 2019: the RCWR was shown to reduce work hours and increase sleep. Further research needs to be accomplished to optimize the shift duration and interval between shifts to allow for sufficient sleep prior to all work shifts.  Landrigan 2020: Contrary to our hypothesis, resident physicians who were randomly assigned to schedules that eliminated extended shifts made more serious errors than resident physicians assigned to schedules with extended shifts, although the effect varied by site. The number of ICU patients cared for by each resident physician was higher during schedules that eliminated extended shifts. during schedules that eliminated extended shifts.  Rahman 2021: Performance impairment due to EDWR is improved by limiting shift duration. These data and their correlation with SME rates highlight the impairment of neurobehavioral performance due to extended-duration shifts and have important implications for patient safety. |
| Bilimoria 2016 [39] | USA | Cluster-randomized, pragmatic, non-inferiority trial- FIRST | 4330 residents | Residents from 117 ACGME accredited general surgery residency programs (87% of the 136 eligible programs) | Standard-policy group compared to flexible policies that waived rules on maximum shift lengths and time off between shifts (flexible-policy group). Standard: 24 to 28-hour shifts, Flexible: PG1 duty periods can exceed 16 hours, PGY 2–5 duty periods can exceed 28 hours. | Flexible duty-hour policies for surgical residents were noninferior to current ACGME duty-hour policies with respect to patient outcomes. Residents’ satisfaction regarding their overall well-being and education quality was similar in the flexible-policy and standard-policy groups. |
| Desai 2018 [48] Silber 2019 [47] Basner 2019 [108] | USA | Cluster-randomized controlled trial - ICOMPARE | 6,313 residents | Internal medicine residents from 63 residency programs | Standard-policy group compared to flexible policies that waived rules on maximum shift lengths and time off between shifts (flexible-policy group). Standard: 24 to 28-hour shifts, Flexible: PG1 duty periods can exceed 16 hours, PGY 2–5 duty periods can exceed 28 hours. | Desai 2018: Compared with a 2003-compliant model, two 2011 duty hour regulation–compliant models were associated with increased sleep duration during the on-call period and with deteriorations in educational opportunities, continuity of patient care, and perceived quality of care. Silber 2019: Allowing program directors flexibility in adjusting duty-hour schedules for trainees did not adversely affect 30-day mortality or several other measured outcomes of patient safety. Basner 2019: This noninferiority trial showed no more chronic sleep loss or sleepiness across trial days among interns in flexible programs than among those in standard programs. Noninferiority of the flexible group for alertness was not established. |
| Landrigan 2004 [68] | USA | Cluster-randomized controlled trial | 24 residents | Medical and Cardiac intensive care unit residents from a single hospital | Work shifts were divided in two: a “day-call” shift from 7 a.m. to 10 p.m. and a “night-call” shift from 9 p.m. to 1 p.m. the following day compared to traditional schedule with extended (24 hours or more) work shifts every other shift (an "every third night" call schedule) | Residents made substantially more serious medical errors when they worked frequent shifts of 24 hours or more than when they worked shorter shifts. Eliminating extended work shifts and reducing the number of hours interns work per week can reduce serious medical errors in the intensive care unit. |
| Parshuram 2015 [61] | Canada | Cluster-randomized controlled trial | 47 residents | Internal medicine, anesthesia, surgery and emergency medicine unit residents from two hospitals | A comparison between: 24-hour schedule (8 am- 8:30 am the next morning followed by 24 hours free of duty), 16-hour schedule (4:30 pm- 8:30 am the next day followed by 24 hours free of duty), and 12-hour schedule (8:30 pm- 8:30 am the next day, worked for 3 or 4 consecutive nights, followed by a 72-hour period free of clinical duties). | We found no significant differences among 3 commonly used resident duty schedules in terms of adverse event rates and residents’ sleepiness. No schedule protected against overnight fatigue or burnout. Our findings do not support the purported advantages of shorter duty and highlight trade-offs between residents’ symptoms and multiple secondary measures of patient safety. More precise quantification will require the conduct of larger randomized studies. |
| Desai 2013 [70] | USA | Cluster-randomized controlled, crossover trial | 43 residents | Residents from four general medical teams in an academic hospital | One of two 2011-compliant models of every fifth night overnight call (Q5) 9 PM-1 PM the next day or a night float (NF) schedule which used day and night shifts with approximately 6 consecutive nights, each with maximal continuous duty of 14 hours and with day shifts the remainder of the study period. Both with 16-hour duty limits. | Compared with a 2003-compliant model, two 2011 duty hour regulation–compliant models were associated with increased sleep duration during the on-call period and with deteriorations in educational opportunities, continuity of patient care, and perceived quality of care. |
| Huber 2020 [106] | USA | Randomized, crossover, mixed-methods trial | 30 residents | Pediatric residents at a post graduate year-two (PGY-2) and post graduate year-three (PGY-3) level at a university medical center | Intervention schedule was 12-hour clinical education days, 6 days per week, with one day off. The shifts were all daytime shifts from 6 am until 6 pm. Comparison schedule was 10- hour clinical education days with a 24 + 4 (28 h) call every fourth night. | There is no difference between the call or shift schedule in regard to residents' perceived knowledge, professionalism, and fatigue. Participants expressed learner preferences for one schedule over the other, recommending the shift schedule during the PGY-2 year and the call schedule during the PGY-3 year. |
| Persico 2018 [107] | France | Randomized, crossover, comparative trial | 21 residents | Emergency department (ED) residents from an 800-bed teaching hospital | Night shift from 6:30 pm to 8:30 am (14 hours) compared to day shift followed by a consecutive night shift (24 hours, from 8:30 am to 8:30 am) | The cognitive abilities of emergency physicians were significantly altered after a 24-hour shift, whereas they were not significantly different from the rested condition after a 14-hour night shift. Limiting 24-hour shift work for emergency physicians should be considered and further evaluated. |
| Barger 2023 [69] | USA | Nationwide, prospective cohort study | 398 residents | Resident physicians for eight academic  years (2002-07  and 2014-17) | Shifts of extended duration (≥24 hours) compared to non-extended duration | Working one or more shifts of extended duration in a month while averaging no more than  80 weekly work hours was associated with an 84%  increased risk of medical errors (1.84, 1.66 to 2.03), a 51% increased risk of preventable adverse events (1.51, 1.20 to 1.90), and an 85% increased risk of fatal preventable adverse events (1.85, 1.05 to 3.26).  Also increased the risk of near  miss crashes (1.47, 1.32 to 1.63) and occupational exposures (1.17, 1.02 to 1.33) were seen. |
| Lazaro 2023 [98] | USA | A retrospective single-program study | 19,393 procedures | Neurosurgery residents from a single-program ( 3 hospitals) | Accreditation Council for Graduate Medical Education 2011 (Night float call from 5:00 pm to 6:00 am) | Junior residents received a concentrated educational experience, whereas senior residents saw a significant  decrease from 112 calls/year to 17. Logged cases significantly increased after implementation of the night float system  (8846 vs 10,547, p = 0.04), whereas cases at non–night float hospitals remained the same.  This difference was mainly driven by senior resident cases (p = 0.010), as junior and  chief residents did not show significant differences in logged cases (p > 0.40). |
