# Supplementary material for: The impact of shortening shifts of physicians during their residency on patients and physicians: A systematic review and meta-analysis
Source: Isr J Health Policy Res. 2025 Sep 3;14:53. doi: 10.1186/s13584-025-00715-2 (PMC12406601; doi:10.1186/s13584-025-00715-2)
Supplement: Supplementary file 4 — Supplementary Material 4 [file 13584_2025_715_MOESM4_ESM.docx]

Supplementary Table 4: Risk of bias assessment for the included observational studies according to the ROBINS-I (Risk Of Bias In Non-Randomized Studies - of Interventions) tool.

| **Study ID** | **Bias due to Confounding** | **Bias in selection of participants into the study** | **Bias in classification of interventions** | **Bias due to deviations from intended interventions** | **Bias due to missing data** | **Bias in measurement of the outcome** | **Bias in selection of the reported result** | **ROBINS-I Total** |
| --- | --- | --- | --- | --- | --- | --- | --- | --- |
| Lockley 2004 | Serious | Serious | Serious | Low | No Info | Serious | Low | Serious Risk |
| Vucicevic 2014 | Moderate | Moderate | Low | Low | No Info | Moderate | Moderate | Moderate Risk |
| Mycyk 2005 | Serious | Serious | Low | No Info | No Info | Serious | Moderate | Serious Risk |
| Jagsi 2006 | Serious | Serious | Moderate | Moderate | No Info | Serious | Moderate | Serious Risk |
| Hanlon 2008 | Serious | Serious | Low | Moderate | No Info | Serious | Moderate | Serious Risk |
| Paul 2012 | Moderate | No Info | Low | No Info | No Info | Moderate | Moderate | Moderate Risk |
| Ouyang 2016 | Serious | Serious | Low | Moderate | No Info | Serious | Moderate | Serious Risk |
| Krug 2017 | Serious | Serious | Low | No Info | Moderate | Serious | Moderate | Serious Risk |
| Yu 2019 | Moderate | Moderate | Low | No Info | No Info | Serious | Moderate | Serious Risk |
| Alsohime 2021 | Serious | Serious | Moderate | No Info | No Info | Serious | Low | Serious Risk |
| Jaradat 2020 | Serious | Serious | Low | No Info | No Info | Serious | Moderate | Serious Risk |
| Jarman 2004 | Serious | Serious | Low | No Info | No Info | Moderate | Low | Serious Risk |
| Breen 2013 | Serious | Serious | Moderate | No Info | No Info | Low | Low | Serious Risk |
| Elbadrawy 2008 | Serious | Serious | Low | No Info | No Info | Low | Low | Serious Risk |
| Gopaldas 2009 | Moderate | Serious | Low | No Info | No Info | Low | Low | Serious Risk |
| Aynardi 2012 | Serious | Serious | Moderate | No Info | No Info | Moderate | Low | Serious Risk |
| Richter 2014 | Serious | Serious | Moderate | No Info | Serious | Serious | Moderate | Serious Risk |
| Shelton 2014 | Moderate | Serious | Low | No Info | No Info | Moderate | Moderate | Serious Risk |
| Kane 2021 | Serious | Serious | Low | No Info | No Info | Low | Low | Serious Risk |
| Churnin 2016 | Serious | Serious | Moderate | No Info | No Info | Low | Low | Serious Risk |
| Schroeppel 2014 | Moderate | Serious | Low | No Info | No Info | Moderate | Moderate | Serious Risk |
| Schumacher 2015 | Serious | Serious | Moderate | No Info | Serious | Serious | Moderate | Serious Risk |
| Vadera 2015 | Moderate | Serious | Moderate | No Info | Low | Moderate | Moderate | Serious Risk |
| Arora 2015 | Moderate | Serious | Moderate | No Info | No Info | Serious | Low | Serious Risk |
| Ripp 2015 | Serious | Serious | Moderate | No Info | Serious | Serious | Moderate | Serious Risk |
| Blencowe 2011 | Serious | Serious | Moderate | No Info | No Info | Low | Low | Serious Risk |
| Laine 1993 | Moderate | Serious | Low | No Info | No Info | Low | Low | Serious Risk |
| Bailit 2004 | Serious | Serious | Moderate | No Info | No Info | Serious | Serious | Serious Risk |
| Blanchard 2004 | Serious | Serious | Moderate | No Info | No Info | Low | Low | Serious Risk |
| Spencer 2005 | Serious | Serious | Low | No Info | No Info | Serious | Moderate | Serious Risk |
| McElearney 2005 | Serious | Serious | Low | Serious | Moderate | Moderate | Moderate | Serious Risk |
| Ferguson 2005 | Serious | Serious | Low | No Info | No Info | Moderate | Low | Serious Risk |
| Bailit 2005 | Serious | Serious | Moderate | Moderate | No Info | Serious | Low | Serious Risk |
| Hutter 2006 | Serious | Serious | Low | Moderate | No Info | Serious | Moderate | Serious Risk |
| Lim 2006 | Moderate | Serious | Low | Low | No Info | Moderate | Low | Serious Risk |
| Short 2006 | Moderate | Moderate | Low | No Info | No Info | Low | Moderate | Moderate Risk |
| Bhavsar 2007 | Serious | Serious | Moderate | No Info | No Info | Moderate | Low | Serious Risk |
| Jagsi 2008 | Serious | Serious | Moderate | Moderate | No Info | Serious | Moderate | Serious Risk |
| Landrigan 2008 | Serious | Serious | Low | Low | Moderate | Moderate | Moderate | Serious Risk |
| Rosenbluth 2013 | Moderate | Serious | Low | No Info | No Info | Moderate | Moderate | Serious Risk |
| DeLaroche 2013 | Serious | Serious | Moderate | No Info | No Info | Serious | Serious | Serious Risk |
| Pepper 2014 | Moderate | Moderate | Low | No Info | No Info | Low | Moderate | Moderate Risk |
| Rajaram 2016 | Moderate | Serious | Moderate | Serious | Moderate | Moderate | Moderate | Serious Risk |
| Anderson 2017 | Serious | Serious | Low | No Info | No Info | Serious | Serious | Serious Risk |
| Watson 2010 | Serious | Serious | Moderate | No Info | No Info | Serious | Moderate | Serious Risk |
| Schenarts 2005 | Moderate | Moderate | Low | Low | No Info | Serious | Serious | Serious Risk |
| Auger 2014 | Moderate | Serious | Low | No Info | No Info | Serious | Low | Serious Risk |
| Lee 2003 | Serious | Serious | Low | Low | No Info | Moderate | Low | Serious Risk |
| Patel MS 2014 | Moderate | Serious | Low | No Info | No Info | Moderate | Moderate | Serious Risk |
| Nomura 2016 | Serious | Serious | Low | No Info | Serious | Serious | Moderate | Serious Risk |
| Smith 2017a | Serious | Serious | Low | Low | No Info | Serious | Moderate | Serious Risk |
| Scally 2015 | Moderate | Serious | Low | No Info | No Info | Serious | Serious | Serious Risk |
| Barden 2002 | Serious | Serious | Moderate | Moderate | No Info | Serious | Low | Serious Risk |
| Feanny 2005 | Serious | Serious | Low | No Info | No Info | Low | Low | Serious Risk |
| Shetty 2007 | Moderate | Serious | Moderate | No Info | No Info | Moderate | Moderate | Serious Risk |
| Salim 2007 | Serious | Serious | Moderate | No Info | No Info | Serious | Serious | Serious Risk |
| Morrison 2009 | Moderate | Serious | Low | No Info | Serious | Moderate | Moderate | Serious Risk |
| Sarff 2009 | Serious | Serious | Moderate | No Info | No Info | Serious | Moderate | Serious Risk |
| Prasad 2009 | Moderate | Serious | Moderate | No Info | Low | Moderate | Serious | Serious Risk |
| Privette 2009 | Serious | Serious | Low | No Info | No Info | Serious | Serious | Serious Risk |
| Rashid 2012 | Serious | Serious | Low | No Info | No Info | Serious | Moderate | Serious Risk |
| Rajaram 2014 | Moderate | Serious | Moderate | No Info | Moderate | Serious | Serious | Serious Risk |
| Liou 2016 | Moderate | Serious | Moderate | No Info | Serious | Moderate | Low | Serious Risk |
| Marwaha 2016 | Moderate | Serious | Low | No Info | No Info | Moderate | Moderate | Serious Risk |
| Liu 2018 | Moderate | Serious | Low | No Info | No Info | Serious | Moderate | Serious Risk |
| Amabile 2021 | Serious | Serious | Moderate | No Info | No Info | Low | Low | Serious Risk |
| Simpson 2020 | Moderate | Serious | Low | No Info | Low | Moderate | Moderate | Serious Risk |
| Mahesh 2014 | Moderate | Moderate | Low | Moderate | No Info | Low | Low | Moderate Risk |
| Damari 2021 | Serious | Serious | Low | No Info | Serious | Serious | Serious | Serious Risk |
| Volpp 2007 | Moderate | Serious | Moderate | No Info | No Info | Moderate | Moderate | Serious Risk |
| Damadi 2007 | Serious | Serious | Moderate | No Info | No Info | Low | Low | Serious Risk |
| Condren 2015 | Serious | Serious | Moderate | No Info | No Info | Low | Low | Serious Risk |
| Gelfand 2004 | Serious | Serious | Low | Moderate | Serious | Serious | Moderate | Serious Risk |
| Zahrai 2011 | Serious | Serious | Moderate | No Info | No Info | Serious | Moderate | Serious Risk |
| Baskies 2008 | Serious | Serious | Moderate | No Info | No Info | Low | Low | Serious Risk |
| Smith 2017 | Moderate | Serious | Moderate | No Info | No Info | Moderate | Moderate | Serious Risk |
| Beltempo 2018 | Serious | Serious | Moderate | No Info | No Info | Low | Low | Serious Risk |
| Crippen 2018 | Serious | Serious | Moderate | No Info | No Info | Moderate | Low | Serious Risk |
| Yaghoubian 2008 | Moderate | Serious | Moderate | No Info | No Info | Moderate | Moderate | Serious Risk |
| Chung 2004 | Serious | Serious | Moderate | Moderate | No Info | Serious | Serious | Serious Risk |
| Poulose 2005 | Moderate | Serious | Moderate | No Info | No Info | Low | Moderate | Serious Risk |
| Kashner 2010 | Moderate | Serious | Moderate | No Info | Moderate | Serious | Moderate | Serious Risk |
| Yaghoubian 2010 | Moderate | Serious | Low | No Info | No Info | Moderate | Serious | Serious Risk |
| Lindbloom 2014 | Serious | Serious | Low | Low | No Info | Moderate | Low | Serious Risk |
| Babu 2014 | Moderate | Serious | Moderate | No Info | No Info | Moderate | Low | Serious Risk |
| Maxwell 2010 | Serious | Serious | Low | Low | No Info | Moderate | Moderate | Serious Risk |
| Smith 2008 | Moderate | Serious | Moderate | No Info | No Info | Moderate | Moderate | Serious Risk |
| Occhino 2011 | Serious | Serious | Low | No Info | No Info | Moderate | Moderate | Serious Risk |
| Markelov 2011 | Serious | Serious | Low | Low | No Info | Low | Low | Serious Risk |
| Volpp 2013 | Moderate | Serious | Moderate | No Info | Moderate | Moderate | Moderate | Serious Risk |
| Scally 2014 | Moderate | Serious | Low | Low | No Info | Serious | Moderate | Serious Risk |
| Hopmans 2015 | Serious | Serious | Moderate | No Info | No Info | Low | Low | Serious Risk |
| Johnson 2018 | Serious | Serious | Low | No Info | Low | Low | Low | Serious Risk |
| Weaver 2020 | Serious | Serious | Moderate | Moderate | Serious | Serious | Moderate | Serious Risk |
| Weaver 2022 | Serious | Serious | Moderate | Moderate | Serious | Serious | Moderate | Serious Risk |
| Stienen 2019 | Serious | Serious | Moderate | No Info | Serious | Moderate | Serious | Serious Risk |
| Salgado 2022 | Serious | Serious | Moderate | No Info | No Info | Serious | Moderate | Serious Risk |
| Durkin 2008 | Serious | Serious | Moderate | No Info | No Info | Low | Low | Serious Risk |
| Jena 2014 | Moderate | Serious | Low | No Info | No Info | Low | Low | Serious Risk |
| Barger 2023 | Moderate | Serious | Moderate | Moderate | Moderate | Serious | Moderate | Serious Risk |
