# Supplementary material for: The impact of shortening shifts of physicians during their residency on patients and physicians: A systematic review and meta-analysis
Source: Isr J Health Policy Res. 2025 Sep 3;14:53. doi: 10.1186/s13584-025-00715-2 (PMC12406601; doi:10.1186/s13584-025-00715-2)
Supplement: Supplementary file 5 — Supplementary Material 5 [file 13584_2025_715_MOESM5_ESM.docx]

Supplementary Table 3: Risk of bias assessment for the included randomized controlled trials according to the Cochrane risk-of-bias 2 tool for randomized trials
